# From 1D Nanofibers to 3D Nanofibrous Aerogels: A Marvellous Evolution of Electrospun SiO_2_ Nanofibers for Emerging Applications

**DOI:** 10.1007/s40820-022-00937-y

**Published:** 2022-09-26

**Authors:** Cheng Liu, Sai Wang, Ni Wang, Jianyong Yu, Yi-Tao Liu, Bin Ding

**Affiliations:** Innovation Center for Textile Science and Technology, College of Textiles, Donghua University, Shanghai, 201620 People’s Republic of China

**Keywords:** SiO_2_ nanofibers, Nanofibrous aerogels, Structural design, Mechanical reinforcement, Multifunctional applications

## Abstract

One-dimensional (1D) SiO_2_ nanofibers (SNFs), one of the most popular inorganic nanomaterials, have aroused widespread attention because of their excellent chemical stability, as well as unique optical and thermal characteristics. Electrospinning is a straightforward and versatile method to prepare 1D SNFs with programmable structures, manageable dimensions, and modifiable properties, which hold great potential in many cutting-edge applications including aerospace, nanodevice, and energy. In this review, substantial advances in the structural design, controllable synthesis, and multifunctional applications of electrospun SNFs are highlighted. We begin with a brief introduction to the fundamental principles, available raw materials, and typical apparatus of electrospun SNFs. We then discuss the strategies for preparing SNFs with diverse structures in detail, especially stressing the newly emerging three-dimensional SiO_2_ nanofibrous aerogels. We continue with focus on major breakthroughs about brittleness-to-flexibility transition of SNFs and the means to achieve their mechanical reinforcement. In addition, we showcase recent applications enabled by electrospun SNFs, with particular emphasis on physical protection, health care and water treatment. In the end, we summarize this review and provide some perspectives on the future development direction of electrospun SNFs.

## Highlights

- The synthetic strategies of electrospun SiO_2_ nanofibers with diverse structures and their three-dimensional (3D) assemblies are reviewed in detail.
- The brittleness-to-flexibility transition of SiO_2_ nanofibers and the means of mechanical strengthening are discussed.
- The multifunctional applications of 3D SiO_2_ nanofibrous aerogels are emphasized, and the challenges and opportunities for their future development are prospected.

## Graphical abstract

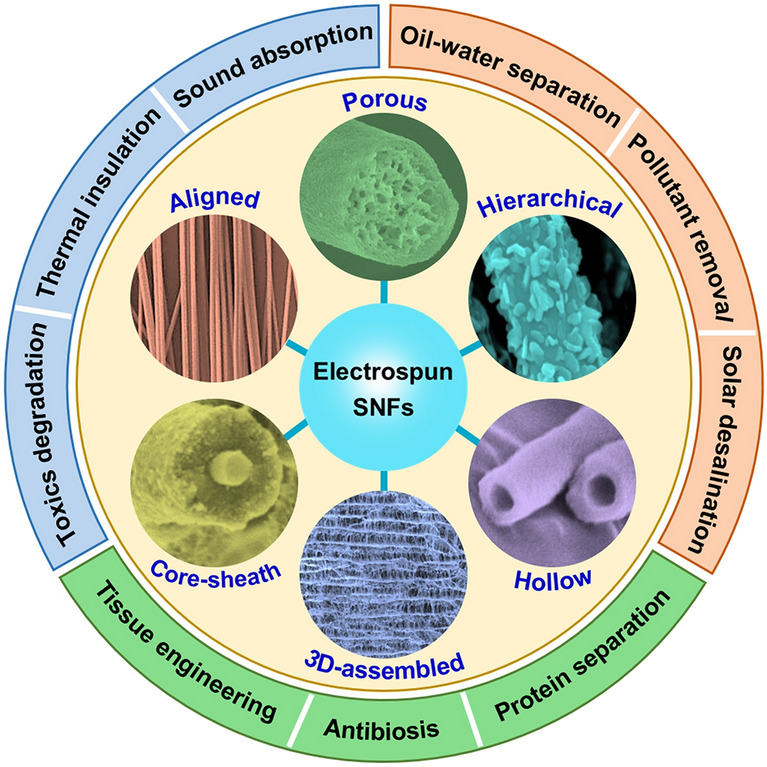

## Introduction

Silica, also known as silicon dioxide (SiO_2_), is a ubiquitous inorganic substance on the planet. The use of SiO_2_ is increasingly important in today’s world. It is no exaggeration to say that SiO_2_ is an essential part of the modern industrial foundation, employed in many industrial areas from glass making to oil extraction: a full list of usages would take up many pages. The significance of SiO_2_ is indisputable, and it is hard to imagine a world in which significant restrictions are imposed on its use [1–3]. Most strikingly, when the dimension of SiO_2_ is reduced to the order of nanometer level, the surface effect and quantum size effect of nanomaterials will give it with unique thermal, optical, mechanical, and electronic properties, which significantly upgrade the performances of ceramic materials in the applications [4, 5]. In fact, Stöber first invented a method in 1968 for synthesizing monodispersed zero-dimensional (0D) SiO_2_ particles with sizes as small as about 50 nm [6]. Since then, researchers have carried out a series of work on this basis and made some progress in the fields of medicine, sensing, and catalysis [7–9]. However, these spherical SiO_2_ nanoparticles are easy to agglomerate, easy to fall off from the substrates, and difficult to recycle, which lay thorny problems for their use. In addition, extremely fine particles are easy to be inhaled by the human body, and their biological toxicity will induce inflammation, tissue lesions, and even organ failure, thus posing serious threats to human health [10, 11]. Therefore, in order to effectively circumvent the above adverse effects, many endeavors have been devoted to the development of desired SiO_2_ nanomaterials with biosecurity, availability, scalability, and practicality.

Compared with 0D SiO_2_ nanoparticles, one-dimensional (1D) SiO_2_ nanomaterials not only have higher surface activity, but also enhance the safety of use owing to their much larger aspect ratio [12]. In the past few decades, various state-of-the-art manufacturing techniques have been explored to prepare different types of 1D SiO_2_ nanomaterials (e.g., nanorods, nanoribbons, nanotubes, nanowires, and nanofibers) [13–15]. Among these techniques, the electrospinning is a simple and versatile approach for producing 1D nanofibers from a wide variety of materials, with representative examples including metals, polymers, ceramics, and organic–inorganic composites [16–18]. Unlike other ways for generating 1D nanomaterials, the nanofibers obtained by electrospinning have obvious advantages in composition control, structure design, and function expansion [19–21]. Moreover, the electrospun nanofibers combine distinctive features such as fine diameter, large specific surface area, and high porosity, thus meeting the needs of diverse applications [22–24]. As for SNFs, in addition to the structural advantages brought by the nanofibers mentioned above, it also has the characteristics of SiO_2_ itself, such as stable chemical properties, high-temperature resistance, low thermal expansion coefficient, high insulation performance, good biocompatibility, and unique optical nature [25–27]. These outstanding comprehensive capabilities endow it with broad application prospects in nanodevices, flexible energy, tissue engineering, and industrial catalysis [28–30]. In 2002, the electrospun SNFs with diameter of 200 ~ 400 nm were prepared for the first time by using polymer/SiO_2_ composite as precursor [31]. Since then, it has aroused widespread research interest and continued to advance the area at an alarming rate. Recent developments in other electrospun ceramic nanofibers can be referred to in many review articles [32–34]. Unfortunately, to the best of our knowledge, there are no comprehensive review articles focusing on electrospun SNFs.

**Fig. 1:**
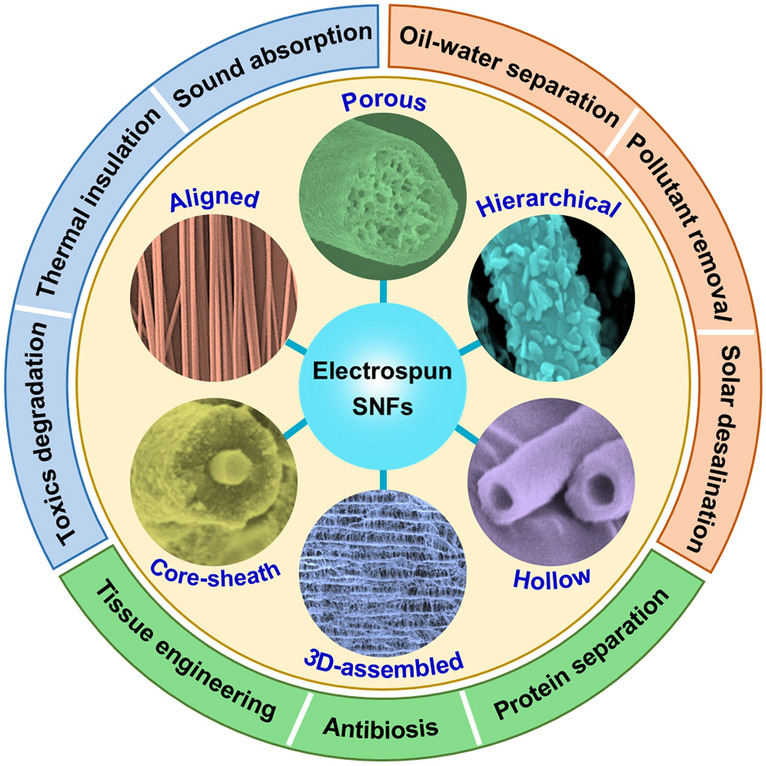
Applications of electrospun SNFs with various structures in many fields. Different SNFs with core-sheath [35], hollow [36], porous [37], hierarchical [38], aligned [39], and 3D-assembled structures [40]. Reproduced with permission from Ref. [35]. Copyright 2013, American Chemical Society. Reproduced with permission from Ref. [36]. Copyright 2009, to the authors (Ming Zhou et al.). Reproduced with permission from Ref. [37]. Copyright 2019, Elsevier. Reproduced with permission from Ref. [38]. Copyright 2014, The American Ceramic Society. Reproduced with permission from Ref. [39]. Copyright 2017, Elsevier. Reproduced with permission from Ref. [40]. Copyright 2018, American Association for the Advancement of Science

In this review, we aim to present an overview of recent progress in electrospun SNFs including design, synthesis, and application (Fig. 1). The text of this article is divided into four parts: In the first part, we will make a brief introduction to the basic principle of preparing SNFs by electrospinning technology; in the second part, we give a comprehensive description of the synthetic strategies of SNFs with different structures, especially the newly emerging three-dimensional (3D) SiO_2_ nanofibrous aerogels; in the third part, we discuss the origin of the excellent flexibility of SNFs and feasible schemes to improve the mechanical properties of SNFs; and in the fourth part, we summarize the advanced applications of SNFs, including special protection, health care, and water treatment. Finally, some personal perspectives on the future development of electrospun SNFs are proposed.

## Fundamentals of Electrospun SNFs

Electrospinning was initially developed as a technology for preparing nanofibers from polymer solutions [41]. By combining solgel chemistry with electrospinning, multifarious organic–inorganic composite nanofibers, and ceramic nanofibers can be obtained [42]. In order to produce well-formed SNFs, a typical process consists of the following three steps: (1) preparation of a stable and homogeneous spinnable solution; (2) fabrication of precursor nanofibers via electrospinning under suitable conditions; and (3) formation of SNFs by calcination at high temperature to remove the organic components [43]. A typical spinnable solution should generally contain a Si precursor, a polymer, a solvent, water, and a catalyst. Si-based metal alkoxides including tetraethyl orthosilicate (TEOS) [44–46] and tetramethyl orthosilicate (TMOS) [47, 48] are often chosen as Si precursors. However, the hydrolysis of TMOS produces methanol, a toxic substance, which is why TEOS is preferred in most cases. Although a few examples of direct electrospinning inorganic sols without polymer addition were noted, the high hydrolysis rates and inapposite rheological properties of such systems present considerable challenges for the control of electrospinning [49, 50]. The employed polymer plays a crucial part in not only adjusting the rheological properties of spinning dope, but strongly affects the morphology and structure of the obtained SNFs [51]. Polyvinyl pyrrolidone (PVP) [52] and polyvinyl alcohol (PVA) [53] are the most widely employed by virtue of their good solubility in water and compatibility with the Si precursor. In addition, a variety of other polymers, such as polyvinyl butyral (PVB) [54], polyacrylic acid [55], polyethylene oxide (PEO) [56], and polyacrylonitrile [57] are also used in some cases. Most notably, given that the Si precursors are based on highly electronegative Si atom, the nucleophilic attack on the central silicon atom by water or hydroxyl group is limited [58]. Therefore, it is necessary to add a proper amount of acid catalyst, such as hydrochloric acid [59], phosphoric acid [60], oxalic acid [61], and acetic acid [62] for accelerating the hydrolysis and condensation reaction.

**Fig. 2:**
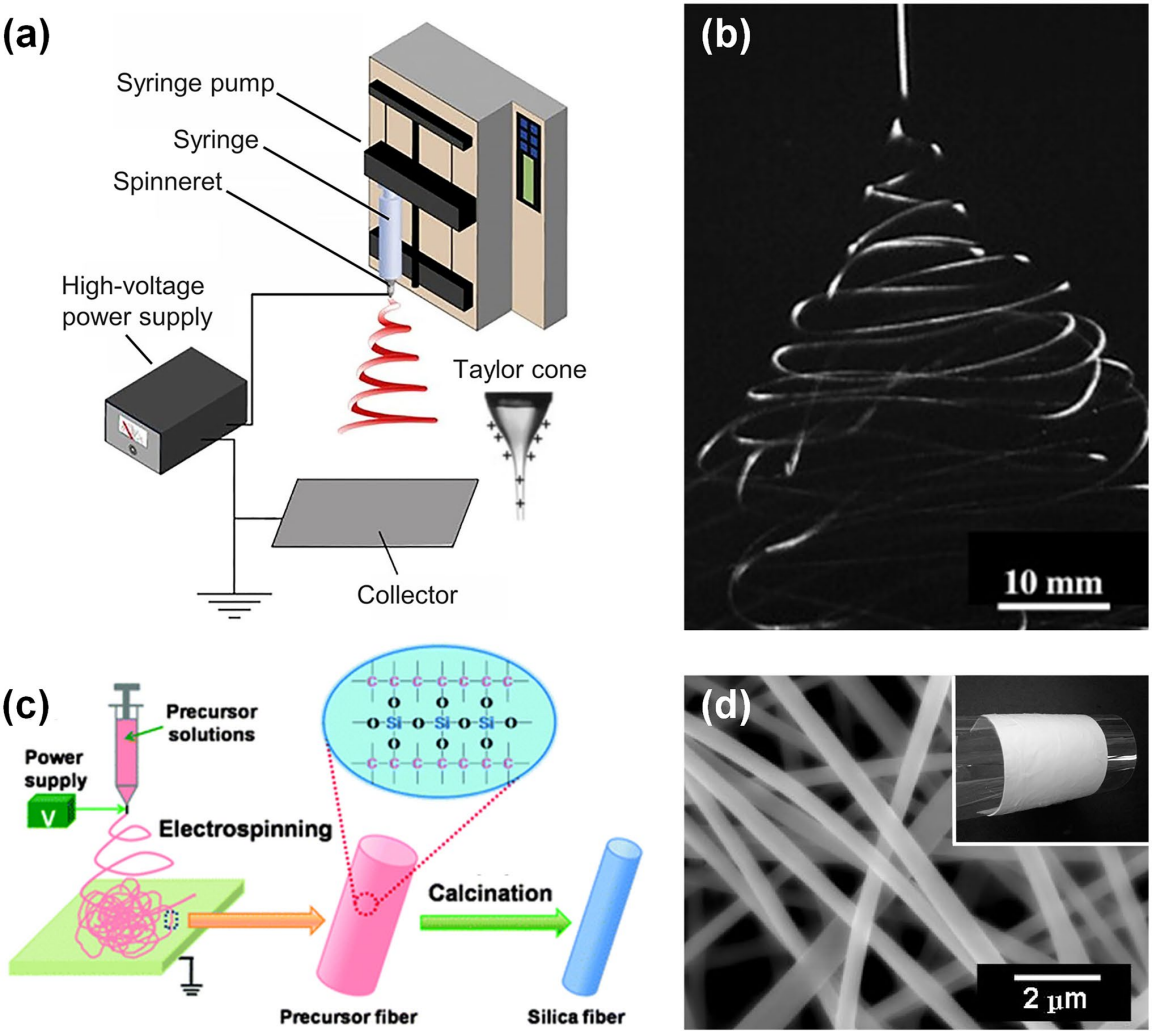
**a** Schematic diagram of basic apparatus for electrospinning [64]. Copyright 2019, American Chemical Society. **b** Photograph of typical jet movement during electrospinning [68]. Copyright 2007, Elsevier. **c** Schematic representation of general process for preparing electrospun SNFs [71]. Copyright 2012, The Royal Society of Chemistry. **d** Scanning electron microscope (SEM) image of SNFs after calcination in air. The inset is the macroscopic flexibility exhibition of SNFs [74].Copyright 2010, American Chemical Society

The equipment of electrospinning is simple and readily available. It generally consists of four sections: a high-voltage direct current power supply, a syringe pump, a spinneret with a small diameter metallic needle, and a grounded collector (Fig. 2a) [63, 64]. In a typical course of electrospinning, the spinning dope is pumped through the spinneret at a controllable speed, and the metallic needle (the inner diameter normally ranges from 0.21 to 1.26 μm) is electrified via the high-voltage power supply. When the solution is squeezed to the tip of the metallic needle, it tends to form a spherical droplet due to the presence of surface tension, but its surface is quickly covered with charges from the applied voltage. The repulsive force of the same sign electric charges competes with the surface tension and makes the shape of the droplet precarious. When the repulsive force is strong enough to exceed the surface tension, the droplet will be transformed into a cone, also known as Taylor cone, and a jet will eject from the tip of the cone. Then the charged jet undergoes an extremely rapid process of bending and whipping, during which it is constantly drafted and elongated toward the collector [65–67]. As illustrated in Fig. 2b, the typical jet photograph has been captured by a camera, demonstrating that the drastic fluctuation and whipping of the jet happen during the electrospinning process [68]. Meanwhile, with the rapid evaporation of the solvent in the spinning process, the diameter of the jet drops sharply and finally solidifies to generate long and thin precursor nanofibers. More strikingly, some crucial parameters affecting the spinning process need to be paid enough attention. For example, the operating parameters (e.g., the applied voltage, the feeding rate of solution, the receive distance, and the motion state of collector) and the environmental parameters (e.g., the temperature and the relative humidity) have a significant impact on the quality of the resultant nanofibers. By optimizing these process parameters, it is possible to obtain nanofibers with desired diameter, arrangement, and morphology [69, 70].

In order to obtain pure SNFs, the as-spun SiO_2_ precursor nanofibers need to be further calcined at high temperature. In the oxidation process of precursor nanofibers, the organic components in the hybrid nanofibers are gradually removed, accompanied by the decrease of nanofiber diameter (Fig. 2c) [71]. In particular, the surface morphology, chemical constitution, crystal structure, and mechanical properties of the SNFs can easily controlled by regulating the calcination parameters (e.g., heating temperature, heating rate, soaking time, and calcination atmosphere) [72, 73]. As an example, Fig. 2d presents the micromorphology of the final SNFs after calcination [74]. Most noteworthy was the flexibility of the SNFs, winding on a PET film without damage, and the excellent flexibility of ceramic nanofibers was demonstrated for the first time. Undoubtedly, it also breaks the traditional perception of brittle nature of ceramic materials and blurs the boundary between polymer materials and ceramic materials. It is the controlled preparation of flexible SNFs that enables the rapid expansion of advanced applications based on them.

## Structure Design of Electrospun SNFs

As well known, materials should be prepared with one or more properties integrated for different application requirements. According to the widely recognized structure–performance relationship in materials science, we need to design the structure of materials more subtly, so that it is more conducive to the functional advantages [75, 76]. So far, a variety of structures have emerged on electrospun SNFs to explore different applications, and they can be grouped into six major categories: core-sheath, hollow, porous, hierarchical, aligned, and 3D-assembled structure.

### Core-Sheath SNFs

The core-sheath structure of nanofibers is an interesting design, realizing the transition from a single-component structure to a multicomponent structure. In addition, due to the highly regulable nature of core-sheath components, it has potential applications in electronic device, drug delivery, and tissue engineering [77–79]. There are two main methods for preparing core-sheath SNFs: One is using coaxial electrospinning method, and the other is employing electrospun nanofiber as template.

#### Coaxial Electrospinning Method

**Fig. 3:**
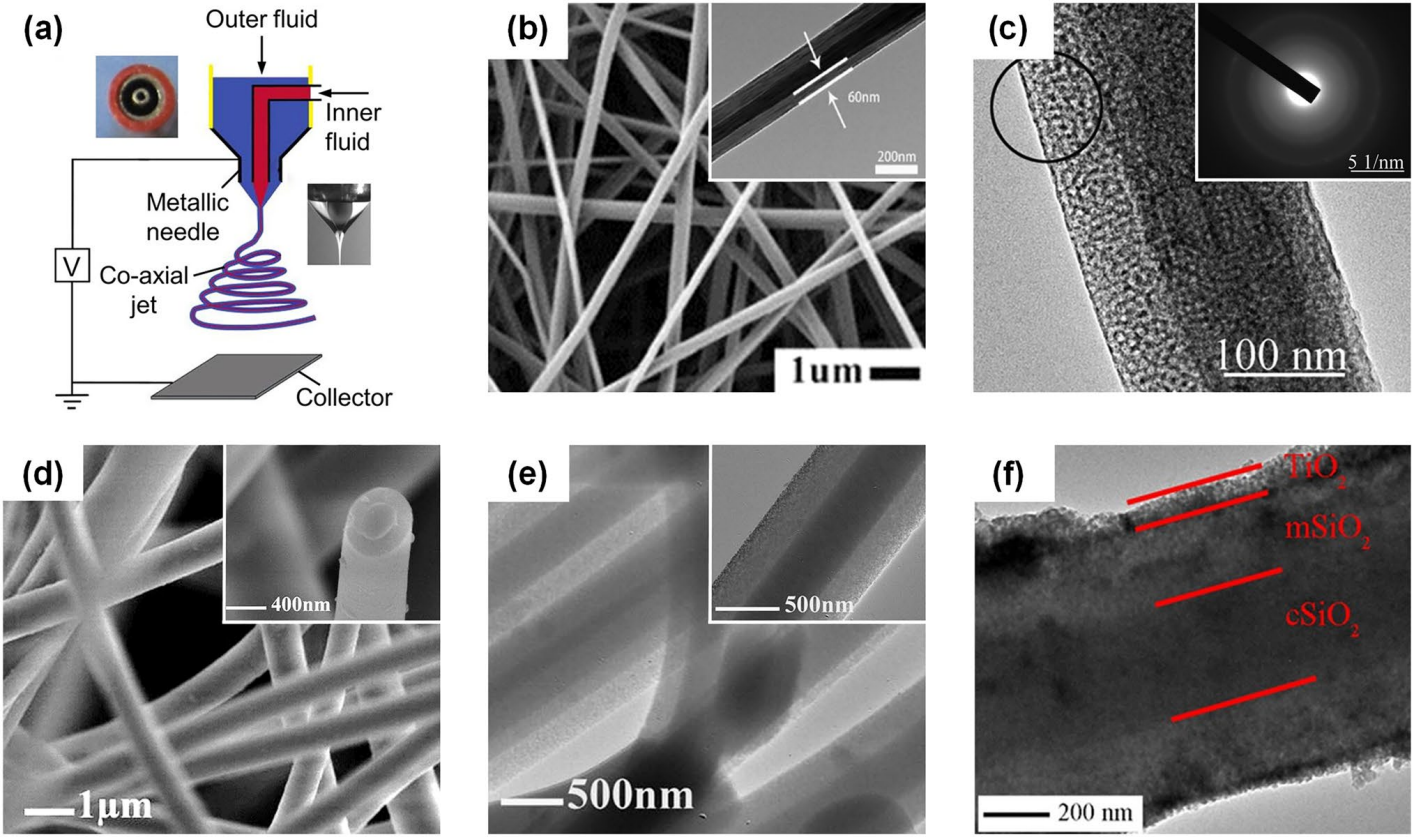
**a** Schematic illustration of coaxial electrospinning device [64]. Copyright 2019, American Chemical Society. **b** SEM photograph of the as-spun TiO_2_/SiO_2_ nanofibers before calcination. The inset is the corresponding transmission electron microscope (TEM) photograph [81]. Copyright 2013, Elsevier. **c** High-resolution TEM photograph of the SiO_2_/Al_2_O_3_ nanofibers after calcination. The inset is the selected area electron diffraction pattern of the part denoted by the circle [82]. Copyright 2014, The Royal Society of Chemistry. **d** SEM image of the SNFs. The inset is the high-magnified SEM image of the corresponding single nanofiber cross section [88]. **e** TEM image of the SNFs. The inset is the magnified TEM image of the corresponding single nanofiber [88]. Copyright 2011, Elsevier. **f** TEM image of the SiO_2_@TiO_2_ nanofibers [89]. Copyright 2014, The American Ceramic Society

Coaxial electrospinning is thought to be an effective and widely used method to prepare core-sheath nanofibers. Figure 3a shows a schematic diagram for coaxial electrospinning. In a typical setup, a coaxial needle including two concentric capillaries was used to generate a coaxial jet during electrospinning [64]. Two viscous liquids are injected into the inner and outer needles at adjustable speeds by two programmable syringe pumps, and the desired coaxial jet can be obtained by applying appropriate voltage to the coaxial needle. Subsequently, after a series of complex processes of stretching, whipping, and solidification, the core-sheath nanofibers with distinct core and sheath composition are formed by the jet [80]. Based on this conventional coaxial electrospinning technique, Cao et al. reported core-sheath TiO_2_/SiO_2_ nanofibers with controlled sheath thickness [81]. They designed TEOS/polyvinyl acetate (PVAc) solution as outer liquid and titanium isopropylate/PVAc solution as inner liquid and finally achieved effective regulation of SiO_2_ sheath thickness by controlling the feeding rate of outer liquid combined with subsequent calcination (Fig. 3b). Moreover, Wang and colleagues also adopted the same spinning method, the difference being that they used SiO_2_ spinnable solution as the core phase and Al_2_O_3_ spinnable solution as the sheath layer phase [82]. After calcination, the synthesized nanofibrous membranes exhibited high strength, which was due to the dense core SNFs that played a decisive role in maintaining mechanical properties (Fig. 3c).

Although the fabrication of core-sheath SNFs by coaxial electrospinning seems simple, the entire implementation process is quite complicated. Many technological parameters must be considered for a successful experiment, especially when a new combining form is required [83, 84]. Herein, we are supposed to pay enough attention to the following points. Firstly, the rheological properties of the core and sheath solutions need to be reasonably regulated to ensure that the liquid jets of the core and sheath can be stretched to the same extent during spinning, leading to the formation of core-sheath nanofibers with high continuity and uniformity. Secondly, it needs to be determined that the core and sheath solution are not miscible, and the solvent of the core cannot be volatile. Otherwise, the solution of the core and sheath will inevitably mix during spinning, so it is difficult to obtain the nanofibers with distinct core-sheath structure. Finally, the thicknesses of the core and sheath of the core-sheath nanofibers should be carefully designed, such as adjusting the feeding rate of the core and sheath solutions or the inner and outer diameter of the coaxial needle, contributing to improving their functionality without compromising mechanical properties as much as possible [85–87].

#### Template-Based Method

Different from coaxial electrospinning method, the template-based method takes the nanofibers prepared by traditional single-needle electrospinning as the core template, and then the core template is post-processed to obtain core-sheath nanofibers. This method is relatively easy to operate without considering the compatibility between different solution systems compared with coaxial electrospinning method.

Based on this principle, Ma et al. reported an intriguing core-sheath SNFs that consisted of a nonporous SiO_2_ core and a mesoporous SiO_2_ sheath [88]. More specifically, the soft nonporous SNFs were fabricated via electrospinning first and then covered by a mesoporous SiO_2_ sheath formed by a modified Stöber method. The SEM and TEM images of the obtained core-sheath SNFs are shown in Fig. 3d-e. Due to the different electronic penetrability between the core and sheath layers, the core-sheath structure can be clearly identified. Based on the study mentioned above, Chen and co-workers took the highly flexible core-sheath SNFs as the skeleton and muffled over with a sheath of TiO_2_ nanoparticles, thus finally forming SiO_2_@TiO_2_ composite nanofibers [89]. As shown in Fig. 3f, the three-layer structure of SiO_2_@TiO_2_ composite nanofibers can be also obviously distinguished by comparison: dense SiO_2_ core layer, mesoporous SiO_2_ intermediate layer, and TiO_2_ sheath layer. Furthermore, Li et al. developed SiO_2_/ZnO nanofibers by combining electrospinning and vapor deposition, in which the ZnO sheath was deposited on the electrospun SNFs by vapor transport [90]. This method could avoid the damage to core nanofibers caused by wet processing to a certain extent and bring forward a new perspective for design core-sheath SNFs.

### Hollow SNFs

Compared to solid SNFs, hollow SNFs have unique advantages in a range of applications such as filtering separation, thermal insulation, and hydrogen storage due to their unique open structure [91]. Two main methods have been adopted to prepare hollow SNFs. One is by sacrificing the template, and the other is by introducing phase separation during spinning.

#### Sacrificial Template Method

The so-called sacrificial template method means that the core-sheath nanofibers are first constructed, and then core component is selectively removed to produce hollow nanofibers. Of course, the construction method of hollow SNFs here is based on the previous section, that is, core-sheath SNFs formed by coaxial electrospinning method or template-based method are post-processed, respectively.

**Fig. 4:**
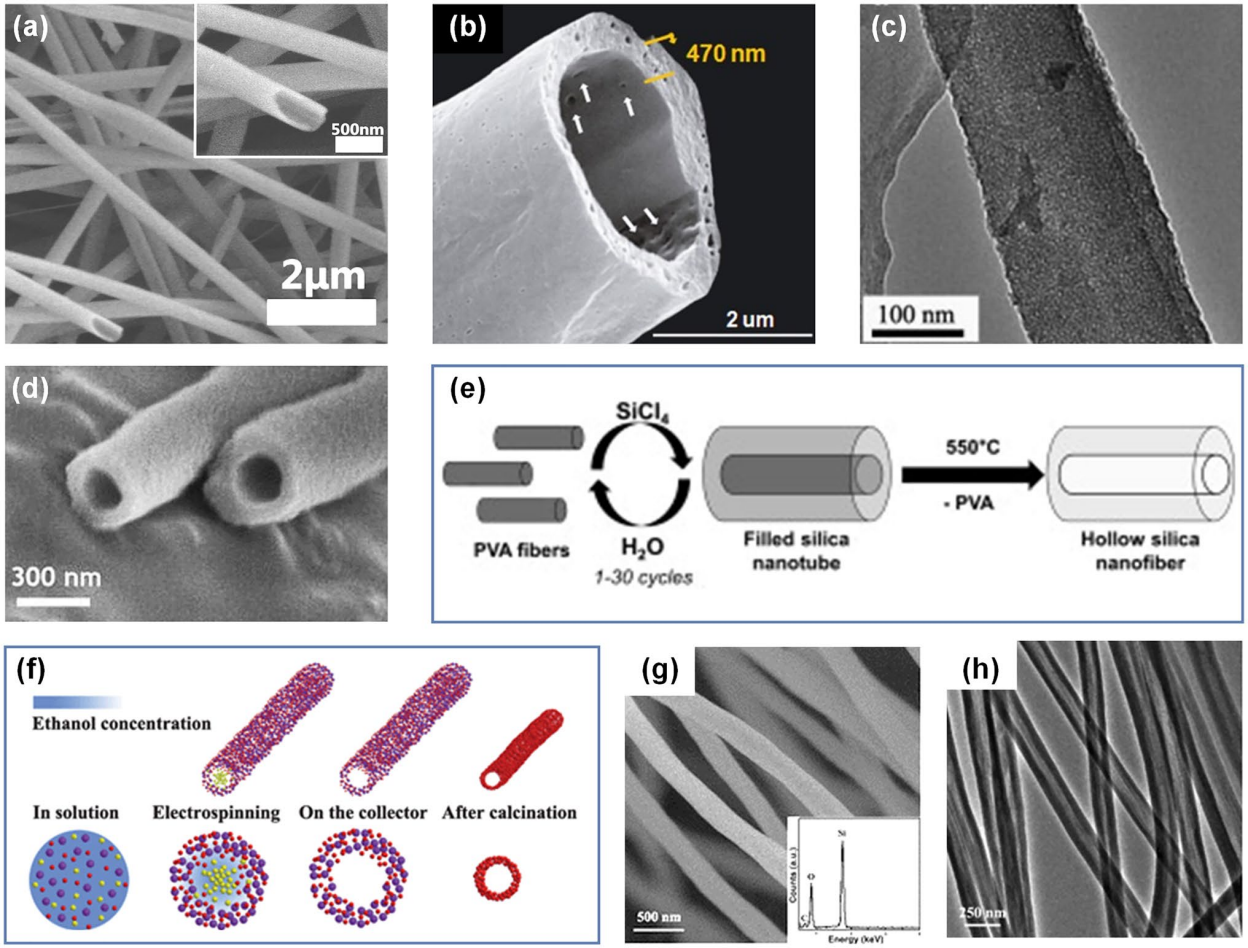
**a** SEM image of hollow SNFs. The inset is the corresponding high magnified SEM image [93]. Copyright 2017, Elsevier. **b** SEM image of hollow SNFs after calcination [94]. Copyright 2011, The American Ceramic Society. **c** High-resolution TEM of hollow SNFs [95]. Copyright 2007, Elsevier. **d** Magnified SEM image of hollow SNFs [36]. Copyright 2009, to the authors (Ming Zhou et al.). **e** Schematic illustration of preparation of hollow SNFs [96]. Copyright 2013, Elsevier. **f** Schematic diagram of the formation mechanism of hollow SNFs [98]. **g** SEM image of hollow SNFs after calcination. The inset is corresponding energy dispersive X-ray spectrum [98]. **h** TEM image of hollow SNFs after calcination [98]. Copyright 2010, The Royal Society of Chemistry

For the core-sheath SNFs prepared by coaxial electrospinning method, generally the core solution is appropriate polymer or mineral oil, which can be easily removed by subsequent high-temperature calcination or extraction [92]. Chen et al. chose TEOS/PVP solution as sheath liquid and polymethylmethacrylate solution as core liquid for coaxial electrospinning [93]. The feeding rate of the solution was optimized during the spinning process, and then the as-spun nanofibers were sintered at 700 °C for 2 h to obtain continuous hollow SNFs with uniform size and smooth surface (Fig. 4a). Moreover, Katoch and colleagues used heavy mineral oil as the core layer and TEOS/PVAc as the sheath layer and then obtained the composite nanofibers by coaxial electrospinning technology [94]. The as-fabricated nanofibers were immersed into octane to remove the mineral oil in the nanofibers and then calcined at 550 °C for 2 h to further remove organic components and residual solvents. Obviously hollow SNFs were formed after calcination, but significant pores were left on the walls of the SNFs due to polymer escape during calcination (Fig. 4b). As we all know, the existence of defects or pores in the nanofibers will have a negative impact on the mechanical properties, let alone these large pores (~ 45 nm) in the hollow SNFs, which is a serious threat. However, Zhan and co-workers found that the hollow SNFs with micropore/mesopore walls were fabricated through coaxial electrospinning [95]. As illustrated in Fig. 4c, the wall of synthesized hollow SNFs presented 3D worm-like porous networks with the homogeneous small mesopores (6 ~ 7 nm). This is caused by the introduction of Pluronic 123 in the sheath inorganic sol, which forms micelles in the system and leads to the formation of worm-like pores.

For the core-sheath SNFs prepared by template-based method, the ultimate purpose is also to remove the core components. Zhou et al. took advantage of the conventional electrospinning technique to prepare well-oriented and ultra-long PVP nanofibers [36]. These PVP nanofibrous arrays were then employed as template to synthesize directional hollow SNFs by plasma enhanced chemical vapor deposition combined with subsequent calcination process (Fig. 4d). It is worth mentioning that the inner diameter and wall thickness of hollow SNFs can be controlled, by simply adjusting the baking time of the polymer nanofibers as well as the coating time of the polymer nanofiber surface without sacrificing orientation degree and array length. Moreover, Müller and colleagues prepared firstly the PVA nanofibers via traditional electrospinning, then wrapped in a thin SiO_2_ sheath through gas phase mineralization, and followed by high-temperature thermal decomposition of PVA core at 550 °C (Fig. 4e) [96]. The hollow SNFs wall thickness was regulated by repeated feeding numbers of SiCl_4_ and H_2_O vapors, and the average wall thickness increased by 0.7 nm per cycle. Different from wet solgel dip-coating process, the preparation of hollow SNFs by vapor phase mineralization of PVA nanofibers proved to be an ingenious method. This method can not only control the pore size and wall thickness independently, but also avoid the undesired fusion of hollow SNFs during wet solgel reaction.

#### Phase Separation Method

Phase separation method has also been employed for the preparation of hollow SNFs [97]. In a typical example, the hollow SNFs were fabricated by a straightforward two-step procedure [98]. Firstly, a partially hydrolyzed PVP/SiO_2_ sol was prepared by precisely regulating the molar ratio of H_2_O to TEOS. The solution was then transferred to a single-needle electrospinning machine, and the hybrid nanofibers were collected with appropriate parameters. Secondly, the as-spun nanofibers were placed in muffle furnace and stabilized at 200 °C for 2 h, then heated to 600 °C and calcined for 3 h, and finally obtained pure hollow SNFs. A possible explanation has been proposed for the formation of hollow SNFs. As illustrated in Fig. 4f, partially hydrolyzed TEOS was obtained by adding insufficient amount of water to the PVP/SiO_2_ system, so the blend solution system was made up of PVP, TEOS, ethanol, and SiO_2_. In the process of electrospinning, the ethanol content in the nanofibrous core was higher than that on the surface due to the rapid evaporation of ethanol with low boiling point. The solubility of TEOS in ethanol was higher than that of PVP in ethanol, so TEOS tends to gather in the center of the nanofiber under the effect of ethanol concentration gradient, while PVP was forced to migrate from the nanofiber core to the outer surface. The core-sheath composite nanofibers with TEOS as the core and PVP/SiO_2_ as the sheath were obtained. It is also noted that the core component TEOS is a volatile liquid, so the nanofibers finally deposited on the receiving substrate were PVP/SiO_2_ composite hollow nanofibers. In this way, PVP can be completely removed from the composite nanofibers after high-temperature calcination, and pure hollow SNFs were obtained (Fig. 4g-h). Based on the phase separation effect, An and colleagues conducted a series of regulation on the volume ratio of TEOS to ethanol in the spinning dope and then prepared hollow SNFs by electrospinning and high-temperature sintering [99]. These results suggest that phase separation method may be one of the most direct and effective methods to prepare hollow SNFs.

### Porous SNFs

In general, the nanofibers obtained by electrospinning are solid structures. However, porous nanofibers are required in many cases because of the significant increase in the specific surface area. The increase in specific surface area will undoubtedly bring greater gains in catalysis, adsorption, filtration, and energy fields [100, 101]. Two approaches have been developed to generate porous SNFs: one by selectively removing components from the nanofibers, and the other by inducing phase separation of the polymer–solvent system during electrospinning.

#### Template Removal Pore-Forming Method

It is a common method for synthesizing porous nanofibers by removing designed components from as-fabricated nanofibers. Such predesigned components, often referred to as hard and soft templates, need to be easily removed without destroying the main nanofibers. Next, we will give typical examples to illustrate these two cases.

**Fig. 5:**
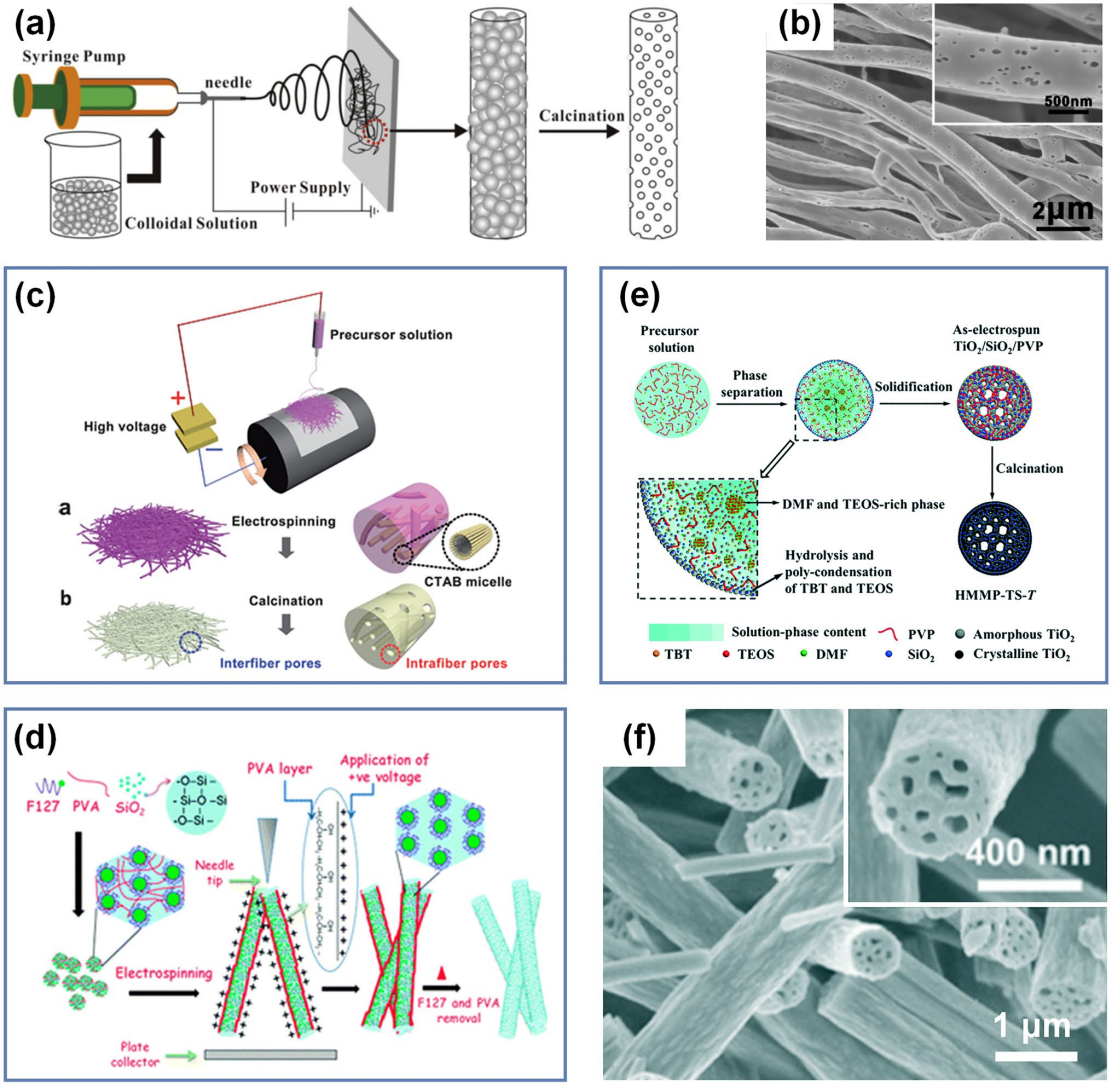
**a** Schematic demonstration of fabrication procedure of the porous SNFs [102]. **b** SEM image of porous SNFs. The inset is the corresponding magnified SEM image [102]. Copyright 2014, Elsevier. **c** Schematic diagram of the preparation process of porous SiO_2_-TiO_2_ composite nanofibers [109]. Copyright 2013, The Royal Society of Chemistry. **d** Proposed mechanism of order mesoporous SNFs [110]. Copyright 2013, The Royal Society of Chemistry. **e** Schematic illustration of the formation process of hierarchically porous TiO_2_-SiO_2_ nanofibers [112]. **f** SEM images of TiO_2_-SiO_2_ nanofibers. The inset is the corresponding magnified SEM image of single nanofiber cross section [112]. Copyright 2017, The Royal Society of Chemistry

Firstly, an example is given to introduce how to realize the controllable preparation of porous SNFs by hard template method. Wu et al. prepared the polystyrene (PS) colloidal dispersions and then added it quantitatively into TEOS/PVP solution to obtain the precursor spinning solution [102]. The PS nanoparticles doped SiO_2_/PVP hybrid nanofibers were generated by conventional electrospinning process. PS and PVP were then removed from the hybrid nanofibers during subsequent high-temperature calcination (Fig. 5a). Here the PS nanoparticles are embedded into the SiO_2_/PVP nanofibers as hard templates, so the finally obtained nanofibers retain the pores that fixed by PS nanoparticles before. As demonstrated in Fig. 5b, this was fully reflected in the surface morphology of the obtained porous SNFs, which possessed a hierarchical porous structure of micropores (~ 3 nm) and mesopores (~ 50 nm). Furthermore, other rigid structural materials with specific shapes, such as SiO_2_/TiO_2_ spheres and carbon spheres, have also been employed as hard templates to prepare porous SNFs [103, 104]. At the same time, it can be noted that the desired pore structure can be controlled by adjusting the size and content of the hard templates [105].

Secondly, porous SNFs can also be prepared by using some materials with no stationary rigid structure but confinement effect in a certain spatial range as soft templates. At present, the developed soft templates mainly include micelles formed by surfactant molecules, microemulsions, polymers, liquid crystals, and biological macromolecules [106, 107]. Among them, surfactants is one of the most common soft templates. It mainly relies on the interaction between surfactant molecules to form micellar aggregates with specific structures (e.g., spheres, rods, and vesicles) in 3D space. Meanwhile, inorganic components are assembled and arranged orderly at the micellar interface, and thus nanomaterials with specific structures can be obtained [108]. Wen and co-workers used cetyltrimethyl ammonium bromide as a soft template to add it into SiO_2_-TiO_2_ blend sol and obtained porous SiO_2_-TiO_2_ composite nanofibers with disordered porous structure after subsequent electrospinning and high-temperature calcination (Fig. 5c) [109]. Remarkably, the specific surface area of the resultant porous SNFs was up to 1032.6 m^2^ g^−1^, the mesoporous volume was 0.46 cm^3^ g^−1^, and the bimodal pore sizes were mainly distributed at 1.1 and 2.2 nm. In addition, Saha et al. reported a “polymer protective layer” strategy to uniformly disperse PVA in TEOS/F127 solution, using the negative charge carried by the hydroxyl group on the PVA molecular chain to neutralize the positive charge applied by the high-voltage power supply (Fig. 5d) [110]. Therefore, a protective sheath was constructed on the outside of TEOS/F127 solution to avoid the damage of internal ordered micelles caused by high charge density during electrospinning. Finally, porous SNFs with highly ordered cubic channels were obtained. It is worth mentioning that the pore size distribution of porous SNFs presents a unimodal and narrow distribution of mesoporous size (~ 5.6 nm), and the large specific surface area is 298 m^2^ g^−1^ without the contribution of micropores. Through these examples, we can find that the porous nanofibers formed by hard template are more dependent on the physical properties of these templates, while the porous nanofibers formed by soft template are more sensitive to the spinning process.

#### Phase Separation Pore-Forming Method

Phase separation in electrospinning, such as non-solvent-induced phase separation and thermal-induced phase separation, has been proved to be an important means to fabricate porous polymer nanofibers [111]. However, a key challenge for preparing porous SNFs is to balance the gelation rate and phase separation rate. Wang and co-workers reported the preparation of porous TiO_2_-SiO_2_ nanofibers by a typical electrospinning process based on a phase separation strategy [112]. They confirmed that the repelling effect between 3D gel network and solvent, hydrolysis polycondensation reaction rate of metal alkoxides, and slow evaporation of high boiling point solvent during electrospinning may be the main reasons for phase separation and finally resulted in the formation of porous SNFs (Fig. 5e). Subsequent analysis found that the pores on the nanofibers showed a hierarchical structure of disordered distribution of mesopores (~ 6.6 nm) and macropores (~ 83.6 nm). As is illustrated in Fig. 5f, macropores can be obviously observed from the morphology of the nanofibers. Furthermore, Wu et al. also prepared TiO_2_/SiO_2_/C nanofibers by electrospinning technique and subsequent carbonization treatment, which had good flexibility and hierarchical pore structure [113]. Due to the rapid evaporation of solvent-rich phase during electrospinning, interconnected pores were left in the precursor nanofibers. In the later carbonization process, the polymer components of the precursor nanofibers were converted to carbon without much effect on the pores that have already formed. Based on the above content, we compare the differences between the two methods in Table 1, hoping to provide some reference for the subsequent design and preparation of porous SNFs. It is not difficult to find that the two methods have their own characteristics. The former usually produces SNFs with high specific surface area, while the latter has advantages in pore size regulation.

**Table 1:** Comparison of preparation methods of porous SNFs

Methods	Polymers	Additives	SSA^*a*^ (m^2^ g^−1^)	Vp^*b*^ (cm^3^ g^−1^)	Dp^*c*^ (nm)	Refs
TRPFM^*d*^	PVP	PS	506	/	3, 50	[102]
	PEO	CTAB	1032.6	0.46	1.1, 2.2	[109]
	PVA	F127	298	0.31	5.6	[110]
PSPFM^*e*^	PVP	TBT^*f*^	29.2	/	6.6, 83.6	[112]
	PVP	TBT	23.2	/	18.5, 93.4	[113]

^*a*^Specific surface area; ^*b*^Pore volume; ^*c*^Pore diameter; ^*d*^Template removal pore-forming method; ^*e*^Phase separation pore-forming method; ^*f*^Tetrabutyl titanate

### Hierarchical SNFs

1D electrospun SNFs have been widely studied in recent years due to their wide availability. Compared with SNFs with a monotonous nanostructure, the nanoarchitecture units with different morphologies and components on the nanofiber surface can not only improve the inherent performance, but also give it some new functional characteristics.

**Fig. 6:**
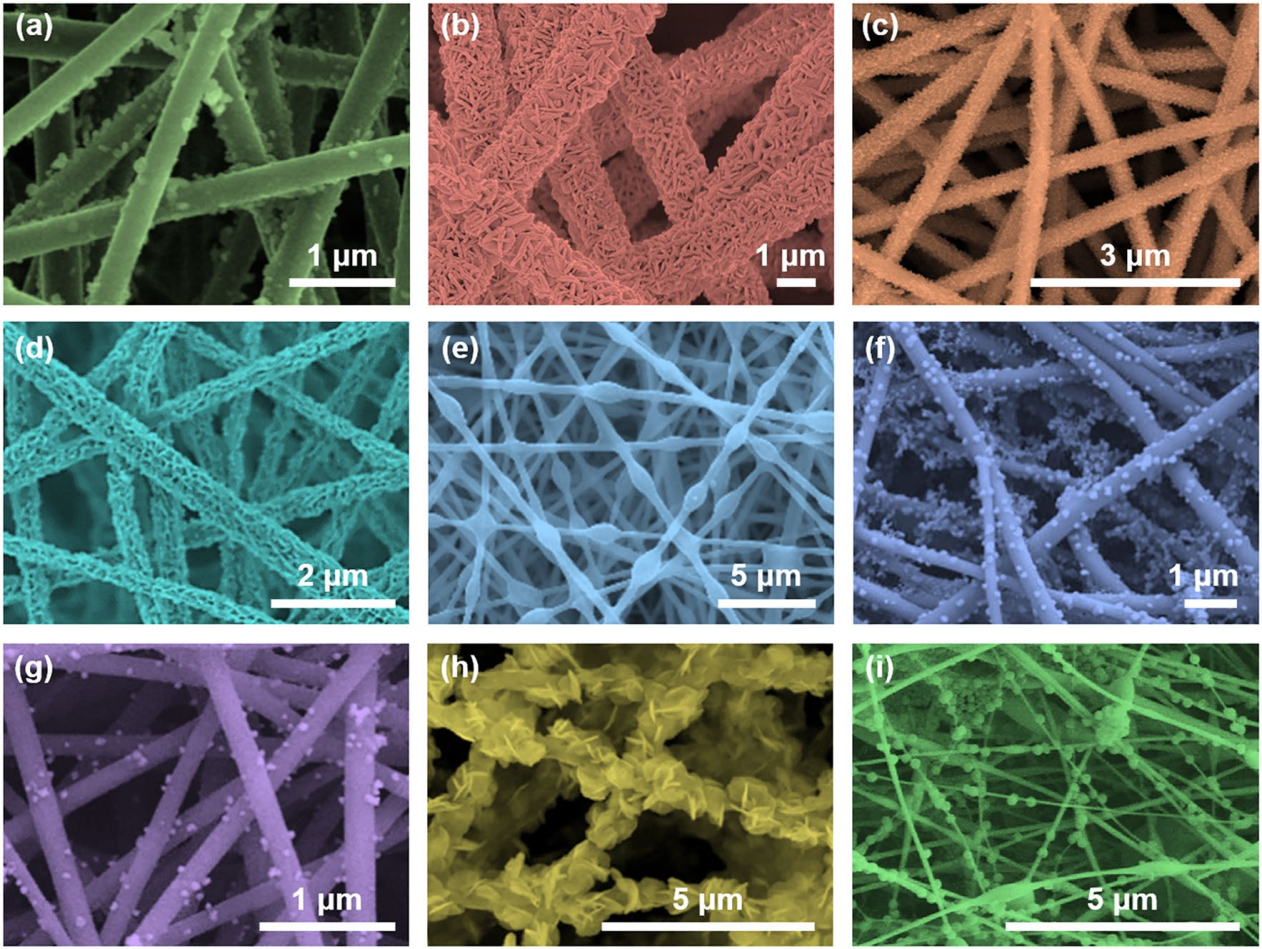
SEM images of hierarchical SNFs: **a** Nickel ferrite nanoparticles anchored onto SNFs [114]. Copyright 2015, American Chemical Society. **b** Boehmite nanoplatelets are anchored on the surface of SNFs [115]. Copyright 2012, American Chemical Society. **c** Polyaniline (PANI) coated on SNFs [116]. Copyright 2017, Elsevier. **d** CuO-ZnO nanosheets deposited on SNFs [117]. Copyright 2022, Springer Nature. **e** PA66 coated on SNFs [118]. Copyright 2021, IOP Publishing Ltd. **f** CuO nanocrystals decorated SNFs [119]. Copyright 2015, Springer Nature. **g** Ag nanoparticles modified SNFs [120]. Copyright 2021, Elsevier. **h** g-C_3_N_4_/BiOI loaded on SNFs [121]. Copyright 2018, Elsevier. **i** ZIF-8 nanocrystals anchored on SNFs [122]. Copyright 2019, Elsevier

Hierarchical SNFs have attracted the attention of many researchers because of their adjustable composition, morphology, and interface. Certainly, the design of hierarchical SNFs mainly involves the construction of nanoscale structural units on the nanofiber surface. These structural units can be roughly divided into the following three categories according to their morphological characteristics: 0D nanostructures (e.g., nanoparticles and nanospheres), 1D nanostructures (e.g., nanowires, nanorods, and nanobelts), two-dimensional (2D) nanostructures (e.g., nanoflakes and nanoplates). The morphologies of some representative examples are presented in Fig. 6. As we can see, secondary structures of various compositions, such as elemental metals, metal oxides, carbides, and metal–organic frames, have been developed [114–122]. In a general way, there are two preparation strategies for these hierarchical SNFs: One is *in situ* growth of secondary nanostructures on the surface of precursor SNFs, and the other is the post-treatment of SNFs in solution to generate secondary nanostructures through chemical reactions or self-assembly.

#### In Situ Growth Method

*In situ* growth is a simple approach to prepare hierarchical SNFs. Over the course of a typical process, the metal salts or metal nanoparticles are added to the spinning dope, and then the as-spun nanofibers are obtained by electrospinning. These introduced additives are finally converted into secondary structures on the nanofibers by high-temperature calcination. For example, Shan et al. prepared Cu-doped C/SiO_2_ nanofibrous membranes through electrospinning and carbonization reduction, and the as-synthesized Cu nanoparticles were uniformly distributed on the C/SiO_2_ nanofiber surface [55]. Wen and co-workers fabricated Pd/SiO_2_ composite nanofibers by a combination of solgel electrospinning, high-temperature calcination, and hydrogen reduction [123]. The synthesized Pd nanoparticles were homogeneously and firmly fixed on the surface of the nanofibers. There is still a lot of room for development of the in situ growth method, which can regulate many factors including the addition of species, calcination atmosphere, and calcination temperature.

#### Liquid Phase Reaction Method

Liquid phase reaction method is one of the most widely used methods for nanomaterials synthesis, which can be combined with electrospinning technology to construct various nanostructure units (e.g., nanospheres, nanorods, and nanoplatelets) on the surface of SNFs. By combining electrospinning with hydrothermal method, Wang and colleagues achieved that a densely distributed layer of MnO_2_ nanosheets covered on the SNFs [124]. It was found that different morphology structures were obtained by adjusting concentration of reactants, and the morphology structures from low to high concentration of reactants were nanowires, nanoflowers, and spike rods. In addition, Hu et al. impregnated SNFs into copper salt solution and obtained SiO_2_-CuO composite nanofibers through subsequent calcination process, on which CuO nanocrystals were deposited [119]. However, this impregnation method, which only relies on physical adsorption, is not conducive to the tight binding of the secondary nanostructures to the nanofiber. Hong and co-workers carried out zein dip-coating process for SNFs, and massive metal ions were loaded in the zein immersion solution [114]. Herein the zein acted as an effective carrier and fixator of metal ions in the subsequent inert gas calcination process. Therefore, the resulting NiFeO_4_ nanoparticles were embedded in the carbon layer and tightly anchored on the SNFs surface. Furthermore, successive ion layer adsorption and in situ polymerization methods have also been developed to prepare various hierarchical SNFs, such as SiO_2_/PANI nanofibers, SiO_2_@g-C_3_N_4_/BiOI nanofibers, and BiOI/SiO_2_ composite nanofibers [116, 121, 125].

#### Other Methods

Unlike the two methods introduced above, which need to go through relatively complicated steps, some simple methods for constructing hierarchical SNFs have also been gradually adopted. For example, Li et al. reported a strategy to synthesize hierarchical SNFs by combining electrospinning and electrospraying technology [126]. Interestingly, the prepared SNFs were composed of nanofibers and beaded structures. In addition, Zhou and colleagues employed SNFs modified by Au nanoparticles as the support carrier and uniformly deposited a layer of g-C_3_N_4_ on the carrier nanofibers by means of vapor deposition. Finally, the desired g-C_3_N_4_/SiO_2_-Au ternary composite hierarchical nanofibers were obtained [127]. There is no doubt that the hierarchical structure formed by these novel methods brings greater benefits to their respective applications. More methods of constructing hierarchical SNFs will be further explored in the future studies, aiming to achieve more efficient applications.

### Aligned SNFs

**Fig. 7:**
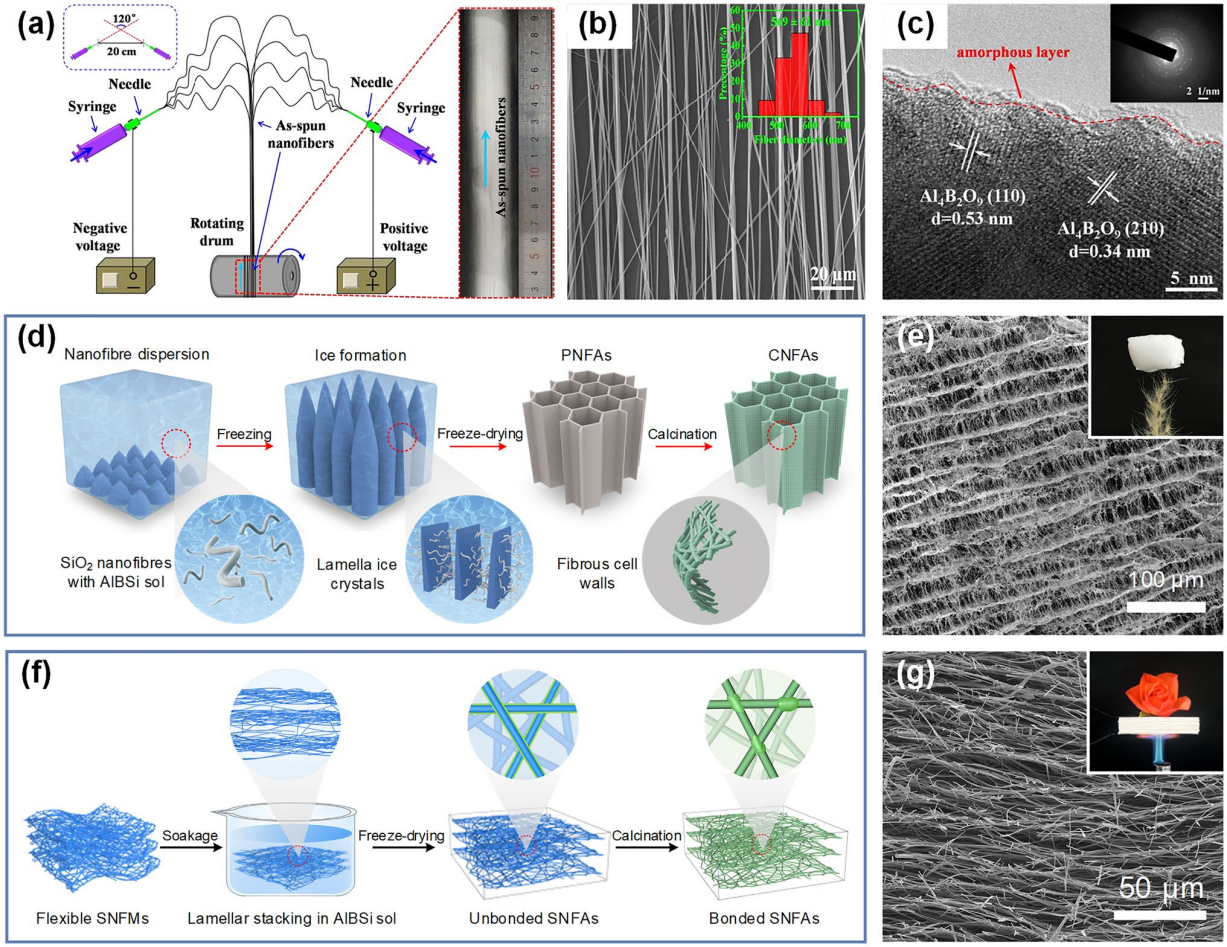
**a** Schematic illustration of conjugated electrospinning apparatus for collecting aligned nanofibers [39]. **b** SEM image of aligned nanofibers after calcination. The inset is the corresponding nanofiber diameter distribution diagram [39]. **c** High-resolution TEM image of aligned nanofibers after calcination. The inset is the corresponding electron diffraction pattern [39]. Copyright 2017, Elsevier. **d** Schematic illustration of the preparation of SiO_2_ nanofibrous aerogels via freeze-drying method [40]. **e** SEM image of the aerogel prepared by freeze-drying method. The inset is an optical photograph showing the ultralight property of aerogel [40]. Copyright 2018, American Association for the Advancement of Science. **f** Schematic presentation of the fabrication pathway of the SiO_2_ nanofibrous aerogels via lamellar stacking method [141]. **g** SEM image of the aerogel prepared by lamellar stacking method. The inset is an optical photograph exhibiting the insulation property of aerogel [141]. Copyright 2021, The Royal Society of Chemistry

In general, the electrospun SNFs are randomly arranged and disordered due to the limitation of bending instability of highly charged jet [128]. Therefore, even after calcination, it does not change its existence in the form of nonwoven mats. However, in many applications, such as electronic energy and optoelectronic devices, electrospun nanofibers with good alignment are particularly needed [129]. Well-aligned nanofibers can be achieved by mechanical, electrostatic, or magnetic methods. Mechanical methods usually involve the use of a high-speed rotating drum or disk collector, causing the nanofibers to be deposited in the direction in which the collector rotates [130]. The electrospun nanofibers can also be aligned by an array of electrodes. By specially designing a pair of electrodes spaced by an air gap to manipulate the external electrostatic field, a uniaxial arrangement of nanofibers along the gap can be obtained [131]. Moreover, a small number of magnetic particles were added into the spinning solution and two parallel permanent magnets were introduced during electrospinning. In this manner, the magnetized nanofibers are driven in a parallel way along the magnetic field lines by an external magnetic field, resulting in directional nanofibers as well [132]. However, there are few reports about the preparation of aligned SNFs by electrospinning. As depicted in Fig. 7a, Song and co-workers fabricated continuous mullite nanofibers composed of Al_4_B_2_O_9_ phase and amorphous SiO_2_ phase by conjugated electrospinning technique [39]. It should be noted that the angle between the two metallic needles assembled on the syringes was 120°, and the distance between the two tips is 20 cm. During electrospinning, a static voltage of + 3.5 and -3.5 kV was applied to the two metal needles, respectively. After calcination at 1000 °C, the nanofibers with the average diameter of 589 nm still maintained favorable alignment and continuous structure (Fig. 7b). In addition, the surface of nanofibers was covered with a thin amorphous SiO_2_ layer by high-resolution TEM micrograph (Fig. 7c). Although this method has achieved good results, the preparation conditions are relatively harsh, and more efficient and easily to implement methods still need to be explored.

### 3D SNFs Assemblies

It is well known that traditional electrospinning can only produce densely deposited 2D nanofibrous membranes with macroscopic thickness of only a few hundred microns or less. There is no doubt that the application of these dense nanofibrous membranes in many fields, such as tissue engineering, filtration, and adsorption, is limited due to their thickness [133]. Electrospun nanofibers are expected to be an ideal building block for 3D nanofibrous assemblies due to their availability, extensibility, and easy regulation [134–136]. At present, there are two main methods to construct 3D nanofibrous assemblies by electrospun nanofibers: one was freeze-drying method first reported by Si et al., and the other was lamellar stacking method recently discovered by Ding’s group [137, 138].

#### Freeze-Drying Method

SiO_2_ aerogel, as a common SiO_2_ monolithic material, has been widely studied since its discovery in the 1930s [139]. Although this kind of aerogel has excellent performance in some physical properties, such as low density, quite transparency, and high porosity, its discontinuous pearl necklace-like intrinsic structure will inevitably lead to a catastrophic event of structural collapse when subjected to high stress or strain [140]. Therefore, Si et al. innovatively prepared ultralight and superelastic SiO_2_ nanofibrous aerogel with a hierarchical and lamellar cellular structure [40]. As is shown in Fig. 7d, the primary pathways for the preparation of SiO_2_ nanofibrous aerogels are as follows: (1) the electrospun SNFs are dispersed into short, fragmented nanofibrous dispersion liquid; (2) the highly homogeneous and dispersed nanofibrous slurry is assembled by cryogenic freezing to form a 3D network structure; (3) the preformed network structure is freeze-dried to sublimate the ice crystal template, and the composite pre-aerogels are obtained; (4) the freshly prepared pre-aerogels are calcined to generate robust cross-linked networks, endowing the finally obtained SiO_2_ nanofibrous aerogels with high elasticity and thermal stability. The SEM image in Fig. 7e showed the lamellar cellular structure of the carefully prepared aerogel, and a piece of 20 cm^3^ of aerogel stood freely on the tip of the feather, further highlighting its ultralight properties. Admittedly, this method for preparing novel aerogels opens the way to the synthesis of many attractive materials. These aerogels play a key role in many fields, which will be detailed in the application section.

#### Lamellar Stacking Method

As we can see, the freeze-drying method for the synthesis of ceramic nanofibrous aerogels combines the versatility of electrospinning with the simplicity of nanofibrous freezing casting, which can be thought as a milestone breakthrough in the development of 3D nanofibrous assemblies. However, it was noted that the nanofibrous aerogels prepared by the above method were assembled by fragmented short nanofibers. Although cross-linking effects were generated between nanofibrous lap joints, such point-to-point forced state could hardly resist strong stress or severe deformation [138]. In this case, Zhang and colleagues reported a novel strategy for fabricating SiO_2_ nanofibrous aerogels [141]. As shown in Fig. 7f, compared with the freeze-drying method, this method employed flexible SNFs as the starting material rather than short nanofiber dispersion liquid. The 3D preformed assembly was prepared by layered stacking of SNFs membranes in impregnation solution, and then the desired SiO_2_ nanofibrous aerogel was finally obtained by subsequent freeze-drying and calcination processes. Different from the nanofibrous aerogels prepared by freeze-drying method, the SiO_2_ nanofibrous aerogels prepared by lamellar stacking method are equipped with multi-arched lamellar structure. These aerogels also showed excellent thermal insulation, which effectively protected flower from wilting in the heat (Fig. 7g). It is because of this special structure that aerogel resisted to external stress in a face-contacting way, thus endowing it robust mechanical strength. Considering that this is a promising new method, it is expected that more novel nanofibrous aerogels with both mechanical strength and functionality will be prepared.

## Mechanical Behavior of Electrospun SNFs

As mentioned above, most of the previous studies focused on the structure design and preparation of electrospun SNFs while ignoring their mechanical properties. However, more attention should be paid to the mechanical properties in the practical use, especially for the mechanical properties of the requirements of the field, such as recyclable catalyst carrier, vibration-resistant insulation sleeve, and water treatment separation membrane. The precursor nanofibers produced by electrospinning are usually xerogel nanofibers and then undergo an essential calcination process to obtain pure ceramic nanofibers. During the calcination process, a series of complicated physical and chemical changes take place in the nanofibers, such as decomposition of organic component, removal of solvent, thermal condensation of metal hydroxyl groups, and movement and rearrangement of atomic. The mechanical properties of the ceramic nanofibers will often deteriorate due to the uneven surface, pore defects, and grain coarsening caused by the above process [142, 143] Therefore, there is no doubt that a deep understanding of the mechanical behavior of electrospun SNFs is of great urgency for the development of materials with excellent mechanical properties to serve advanced applications.

### Origin of Flexibility in SNFs

**Fig. 8:**
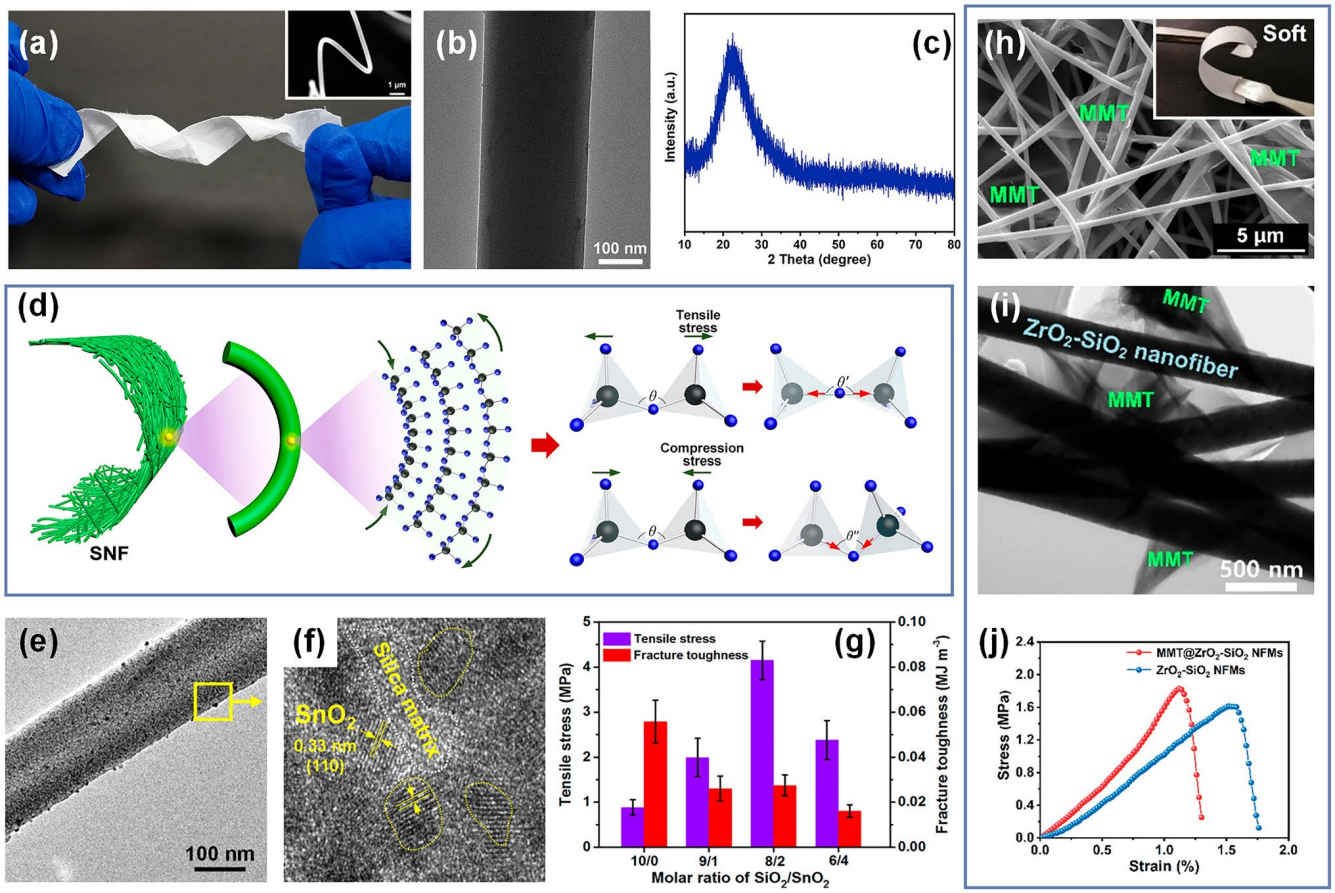
**a** Optical photograph of a flexible SNFs membrane. The inset is the SEM image of the corresponding flexible single nanofiber [147]. **b** TEM image of the SNFs [147]. **c** XRD spectrum for the SNFs [147]. **d** Plausible mechanism for the flexibility of SNFs [147]. Copyright 2022, American Chemical Society. **e** TEM image of the SiO_2_/SnO_2_ nanofiber [161]. **f** High-resolution TEM image of a selected area [161]. **g** Tensile strength and fracture toughness of SiO_2_/SnO_2_ nanofibrous membrane [161]. Copyright 2017, American Chemical Society.**h** SEM image of the MMT@ZrO_2_-SiO_2_ nanofibers. The inset is the corresponding optical image of soft nanofibrous membrane [164]. **i** TEM image of MMT@ZrO_2_-SiO_2_ nanofiber [164]. **j** Stress–strain curves of ZrO_2_-SiO_2_ and MMT@ZrO_2_-SiO_2_ nanofibrous membranes [164]. Copyright 2021, American Chemical Society

In 2002, Kim’s group prepared SNFs by electrospinning for the first time, which paved the way for the manufacture of other inorganic nanofibers [31]. Subsequently, TiO_2_, ZrO_2_, and Al_2_O_3_ nanofibers have also been developed by this method [144–146]. However, these ceramic nanofibers basically showed the inherent brittleness characteristics of ceramic materials, which greatly reduced their use value. Until 2010, Ding’s group prepared flexible SNFs for the first time, but the flexibility mechanism of the nanofibers was not fully explained [74]. Recently, Cao and co-workers accurately controlled the composition of SiO_2_ sol and the amount of PVA and prepared electrospun SNFs with excellent flexibility [147]. It was observed that the synthesized SNFs membrane could be twisted and bent macroscopically without any damage. Meanwhile, the single nanofiber at the microscale was also subjected to large bending deformation without brittle fracture, which fully demonstrated the superior flexibility of the SNFs (Fig. 8a). In order to further analyze the flexible mechanism of SNFs, they also studied the microstructure and crystal structure in detail. They found that the prepared SNFs were amorphous and no obvious grains were found, which was further confirmed by the X-ray diffraction pattern (Fig. 8b-c). On this basis, a reasonable explanation for the remarkable flexibility of amorphous SNFs was proposed. The amorphous SiO_2_ is considered as a continuous random network of relatively flexible SiO_4_ tetrahedrons. The Si–O-Si bond angles for amorphous SiO_2_ exbibit a broad distribution from 120° to 180° and mainly concentrated at 144° [148, 149]. When the single nanofiber was subjected to external bending stress, the Si–O-Si bonds on the outer side of the nanofiber surface were stretched and the bond angles increased. At the same time, the Si–O-Si bonds on the inner side of the nanofiber surface were compressed, and the bond length and bond angle decreased (Fig. 8d). Therefore, it is the switchable bond lengths and bond angles of Si–O-Si bonds in the silicon oxygen tetrahedron network that may endow SNFs surprising flexibility.

### Strengthening Strategies for SNFs

At present, the preparation of flexible SNFs is no longer a thorny problem, and the further application of it is often reported. However, we clearly realize that the tensile strength of the current electrospun SNFs is still low, which is difficult to meet the needs of practical application. Moreover, there is a strong ionic bond or covalent bond inside the ceramic, it is not easy to slip when impacted by external forces, and it is difficult to plastic deformation to offset part of the stress [150, 151]. Therefore, it is of great significance to strengthen ceramic nanofibers based on understanding the mechanical behavior of electrospun ceramic nanofibers.

A great deal of studies showed that the mechanical properties of oxide ceramic nanofibers largely rely on the interatomic bonding strength, microstructure, and surface morphology [152–154]. Specifically, the pore structures, crystal forms, grain size, and crystallinity of oxide ceramic nanofibers determine the microcrack propagation and stress distribution, which have a momentous effect on the mechanical properties of the nanofibers [155]. It is well known that the distribution of fine grains in nanofibers can increase the proportion of grain boundaries, so that the concentrated stress can be effectively dispersed [156]. Electrospun SNFs are usually amorphous at conventional calcination temperature (800 °C), so the SiO_2_ phase is also often used as a doping component which were introduced to other ceramic materials which crystallized easily. The added SiO_2_ phase inhibits grain growth, leading to multiphase ceramic nanofibers with a small grain size that exhibits a certain degree of flexibility at the macroscale [157–160].

Based on the above understanding of the mechanical behavior of ceramic nanofibers, some works were carried out to improve the mechanical properties of SNFs. It is acknowledged that the mechanical properties of macroscopic materials are closely related to the mechanical behavior of the underlying basic building units. Therefore, it is necessary to study the mechanical properties of single ceramic nanofiber. However, it should be admitted that the mechanical testing of single fiber at the nanoscale is a major challenge, especially the process of preparing single nanofiber samples, so that most of the current works were limited to the mechanical properties of nanofibrous assemblies (e.g., 2D nanofibrous membranes and 3D nanofibrous aerogels). In short, how to strengthen SNFs needs to be considered from the following aspects: (1) structural design; (2) interfacial interaction; (3) preparation process.

In terms of structural design, Shan et al. ingeniously designed a dual-phase ceramic nanofiber, which was embedded in amorphous SiO_2_ nanofiber matrix by SnO_2_ crystal phase [161]. As can be seen from TEM micrograph in Fig. 8e-f, the SnO_2_ fine nanocrystals were randomly distributed in the nanofiber and surrounded by amorphous SiO_2_ phase, which further proved the reliable preparation of dual-phase nanofibers. In fact, the obtained nanofibers can also be taken as classic brick–mortar structures by embedding SnO_2_ nanocrystals (bricks) into the amorphous region of SiO_2_ (mortar). Meanwhile, it can be observed from Fig. 8g that the tensile stress of the resultant nanofibrous membrane was up to 4.15 MPa with appropriate nanocrystalline doping, which was more than 3 times higher than that of the pure SNFs membrane (0.89 MPa). Unfortunately, there is no in-depth explanation for this phenomenon in their publication. It is reasonable to speculate that this may be due to the heterogeneous distribution of nanocrystals in the amorphous matrix which restricts the stable development of the budding shear band. Even if some shear bands are present, they are blocked when they encounter fine grains and subsequently divide into several germinated shear bands. These embedded nanocrystals can slide and rotate only when high enough stress is applied, which effectively overcome the problem of instability fracture of amorphous materials caused by softening effect and extension of shear bands, and ultimately enhancing the mechanical properties of amorphous materials [162, 163].

In addition to improving the mechanical properties of nanofibers from the perspective of single nanofiber structure design, it is also an effective strategy to form stable and strong interface interaction between nanofibers. Mao and co-workers impregnated the as-prepared ZrO_2_-SiO_2_ nanofibrous membranes in the montmorillonite (MMT) dispersion solution and successfully constructed cross-linked MMT nanosheets between ZrO_2_-SiO_2_ nanofibers after subsequent calcination [164]. As is exhibited in Fig. 8h, the cross-linked characteristic between nanofibers were confirmed, which resulted from in situ heat treating of MMT nanosheet on the nanofiber surface. Furthermore, the TEM image (Fig. 8i) showed that MMT nanosheets deposited well on the surface of ZrO_2_-SiO_2_ nanofibers, highlighting the robust interfacial bond between the MMT nanosheets and the ZrO_2_-SiO_2_ nanofibers. As shown in Fig. 8j, benefiting from the cross-linked assembly of nanofibers and nanosheets, the tensile stress of the MMT@ZrO_2_-SiO_2_ nanofibrous membranes (1.83 MPa) was higher than that of pure ZrO_2_-SiO_2_ nanofibrous membranes (1.61 MPa). Moreover, the notable results can be obtained by improving the preparation process of electrospun SNFs. Zhang and colleagues found that ball milling of spinning sol and bending drafting of precursor nanofibers significantly improved the molecular structure order and reduced pore defects of precursor nanofibers, effectively enhancing the tensile strength of SNFs membranes [165]. In general, the above methods have made remarkable progress in the mechanical enhancement of SNFs, but there is still a certain gap from our expected goal. Fortunately, some clear strategies have emerged, and more time and effort will ensure the reliable use of advanced SNFs in terms of mechanical properties.

## Applications of Electrospun SNFs

Electrospun SNFs, as one of the most widely used 1D ceramic nanostructured materials, possess various predominant features including high porosity, large specific surface area, and unique optical properties, which tremendously enhance the performance of their nanofibrous assemblies and greatly widen their application sphere. As we know, SiO_2_ nanofibrous assemblies, especially 3D nanofibrous aerogels, are more convenient to deploy in many application scenarios and give full play to their performance advantages. In this section, we primarily focus on SiO_2_-based nanofibrous aerogels related applications in physical protection, health care, and water treatment, and a lot of innovative works have been done in these crucial research areas in recent years.

### Physical Protection

#### Thermal Insulation

SiO_2_ is a nontoxic and hard inorganic material widely distributed in nature. It is widely used in the field of heat insulation due to its characteristics of good refractory, low thermal conductivity, and stable chemical properties. Compared with the traditional SiO_2_ aerogel insulation material with brittleness and poor mechanical properties, electrospun SNFs insulation material has obvious advantages such as good toughness, high porosity, and high thermal resistance, which has become a research hot spot in this field in recent years. Zheng et al. prepared hybrid SNFs/SiO_2_ aerogel membranes by impregnating SNFs with SiO_2_ sol, then drying at room temperature [166]. The obtained hybrid membranes exhibited enhanced mechanical strength (more than 200% increase in tensile strength) and low thermal conductivity (0.021 W m^−1^ k^−1^). But the material produced in this way still existed as a thin membrane, rather than as a block. Subsequently, Zhang and colleagues successfully synthesized SNFs reinforced SiO_2_ aerogel composites by adding flexible SiO_2_/SnO_2_ nanofibers into SiO_2_ sol and subsequent solgel method and supercritical drying process [167]. Compared with traditional granular SiO_2_ aerogel, the composites are equipped with decreased thermal conductivity from 0.034 to 0.025 W m^−1^ k^−1^ and improved Young’s modulus from 35 to 70 kPa. This is because the added SNFs were well dispersed in the aerogel and bonded with the SiO_2_ aerogel nanoparticles, enabling the skeleton of the aerogel composites to withstand strong capillary force in the supercritical drying process.

Although the mechanical properties of nanofiber-reinforced SiO_2_ aerogel have been improved, it is still difficult to meet the application requirements; especially, the resilience is not satisfactory. Si et al. created superelastic ceramic nanofibrous aerogels by freeze-drying method to assemble random-deposited SNFs into elastic nanofibrous aerogels [40]. The as-fabricated SiO_2_ nanofibrous aerogels showed the comprehensive properties of complete recovery at large compression strain, ultra-low density, and good fire resistance. This work provided valuable reference for the preparation of more ceramic nanofibrous aerogels, especially around the topic of improved the thermal insulation performance. Using a similar approach, Dou and co-workers prepared SiO_2_ nanofibrous aerogels with low thermal conductivity (0.02327 W m^−1^ k^−1^) by combining SiO_2_ nanoparticle aerogels with SNFs [168]. The analysis showed that adding SiO_2_ nanoparticle aerogels can not only reduce the solid thermal conductivity by increasing the solid conduction path, but also reduced the gas thermal conductivity by filling the large pores on the cell wall of the nanofibrous aerogel. Furthermore, Dou et al. assembled nanoporous SiO_2_ particle aerogel in a cellular SiO_2_ nanofibrous framework by dip-coating SiO_2_ nanofibrous aerogel with SiO_2_ sol [169]. The introduction of SiO_2_ particle aerogel formed SiO_2_ particle networks with small pore size in the cellular nanofibrous framework, whose pore size (~ 4 nm) is less than the mean free path of air molecules (~ 75 nm), effectively inhibited the heat conduction of gas, so the thermal conductivity can be as low as 0.02196 W m^−1^ k^−1^.

A series of SiO_2_ nanofibrous aerogels through freeze-drying method have been prepared, which have excellent thermal insulation properties, but are not yet satisfactory in their mechanical properties. This was mainly because the nanofibrous aerogel framework was assembled by short nanofibers in a point-to-point manner, which led to the limitation of its effective force area and difficulty in resisting large external stresses. Zhang and co-workers prepared lamellar multi-arch structured SiO_2_ nanofibrous aerogel by lamellar stacking method [141]. This special structural design enabled aerogels to share the stress face-to-face during the loading process, thus maximizing the strength of the material. Therefore, the compressive strength of the obtained SiO_2_ nanofibrous aerogel can reach 160 kPa under 60% recoverable strain. As a significant contrast, SiO_2_ nanofibrous aerogel prepared by freeze-drying method had a compressive strength of only about 10 kPa under 60% recoverable strain. The mechanical properties of the aerogel were significantly improved by this method, but the thermal conductivity of the aerogel was relatively high at 0.0389 W m^−1^ k^−1^ due to the relatively close stacking between the nanofibrous membranes, so there is still room for improvement in the thermal insulation performance.

**Fig. 9:**
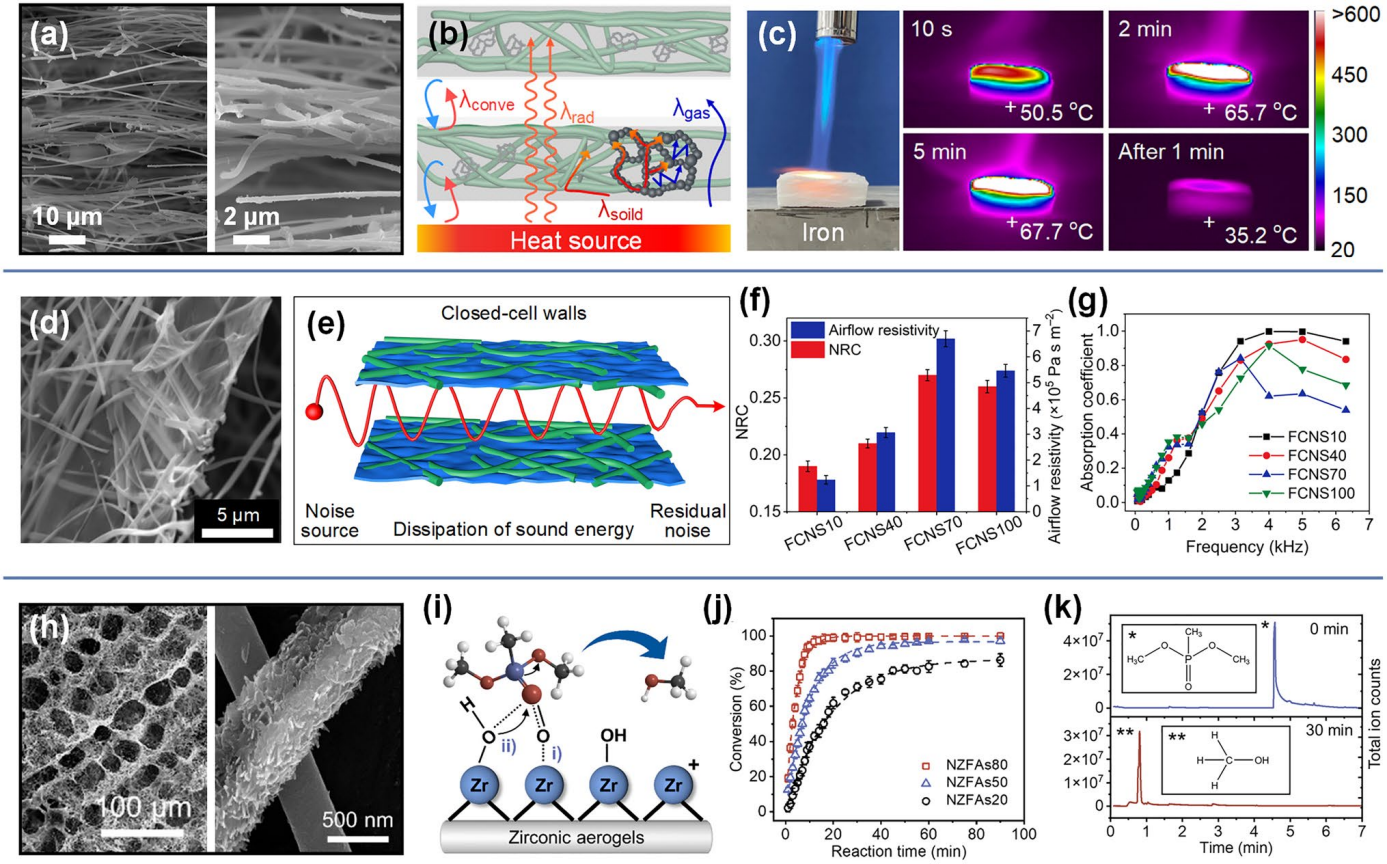
**a** SEM image of ZrO_2_-SiO_2_ nanofibrous aerogel showing its hierarchical structure [171]. **b** Schematic demonstration of factors contributing to thermal conductivity of the ZrO_2_-SiO_2_ nanofibrous aerogel [171]. **c** Optical and infrared thermal images showing temperature distributions of the aerogel jetted by a butane flame [171]. Copyright 2022, American Chemical Society. **d** SEM image of ceramic nanofibrous aerogels exhibiting the hierarchically entangled networks [172]. **e** Schematic illustration of the sound absorption mechanism for closed cell walls [172]. **f** Effects of GO loading amount on noise reduction coefficient (NRC) and airflow resistance of the aerogel [172]. **g** Variation of the absorption coefficient of the relevant aerogels [172]. Copyright 2021, Springer Nature. **h** SEM image of Zr(OH)_4_@PVB/SiO_2_ nanofibrous aerogel showing its hierarchical structure [174]. **i** Mechanism of DMMP degradation by aerogel [174]. **j** Plots of DMMP conversion versus reaction time [174]. **k** Extracted chromatograms for the initial and 30 min DMMP challenges the aerogel. The inset is the molecular formula of the chemical substance of labeled peaks [174]. Copyright 2021, American Chemical Society

On this basis, Zhang et al. finally prepared fluffy and lamellar arched ZrO_2_-SiO_2_ nanofibrous aerogels by stacking ZrO_2_-SiO_2_ nanofibrous membranes layer by layer in the sol and further added a step of ultrasonic processing [170]. As a result of the ultrasonic disintegration effect, the dense ZrO_2_-SiO_2_ nanofibrous membranes were loose in the solution, which was crucial for the formation of highly fluffy nanofibrous aerogel in the subsequent ice-template process. The thermal conductivity of ZrO_2_-SiO_2_ nanofibrous aerogel was as low as 0.0268 W m^−1^ k^−1^ due to the low density and high porosity brought by the highly fluffy structure. However, the micron-sized pores between the nanofibers in these aerogels did not mitigate the heat transfer of the air, so the insulation performance was not as good as expected. Therefore, Zhang and colleagues further impregnated ZrO_2_-SiO_2_ nanofibrous membranes into SiO_2_ sol containing SiO_2_ nanoparticle aerogels and then obtained the ceramic nanofibers–nanoparticles composite aerogels through the ultrasound-assisted ice-template shaping process [171]. Benefiting from the lamellar, multi-arched, and leaf-like nanofibrous-granular binary networks of the novel nanofibrous aerogels, the thermal conductivity of ZrO_2_-SiO_2_ nanofibrous aerogels was significantly reduced to 0.024 W m^−1^ k^−1^. Several key factors were found to be responsible for the excellent heat-shielding performance. One was that the introduction of SiO_2_ nanoparticle aerogels into the porous nanofibrous framework not only increased the solid thermal conduction path, but also locked more air into the nanopores, thus reducing the heat conduction of both solid and gas. The other was that the porous framework and arched structure of aerogel can effectively inhibit the heat conduction and heat convection of gas in the vertical lamellar direction (Fig. 9a-b). As a proof of concept, an ZrO_2_-SiO_2_ nanofibrous aerogel with a thickness of 10 mm was placed on an iron block and exposed to a butane flame. After 5 min, the temperature at the bottom of the aerogel was only 67.7 °C, demonstrating excellent thermal insulation performance (Fig. 9c).

In summary, the application of electrospun SNFs in thermal insulation field has been widely studied and some gratifying progress has been made. For electrospun SiO_2_ nanofibrous materials that have been reported for thermal insulation, we summarize a Table 2 to better demonstrate their thermal insulation properties. With the continuous development of human civilization, the thermal insulation materials also put forward new major challenges. For example, the national defense industry, aerospace, and civil industries have special requirements for nanofibrous aerogel insulation materials with anti-vibration shock, high strength, compressibility, stretchability, bendability, and other excellent properties, which have become an important direction for guiding the research of nanofibrous aerogel insulation materials in the future.

**Table 2:** Comparison of properties of electrospun SiO_2_ nanofibrous materials for thermal insulation

Samples	Maximum working temperature (°C)	Thermal conductivity (25 °C, W m^−1^ k^−1^)	Refs
SiO_2_/C NFAs^*a*^	350	0.023	[26]
SiO_2_ NFAs^*b*^	1100	0.025	[40]
SiO_2_ NFAs^*c*^	1100	0.024	[46]
SiO_2_ CNFs^*d*^	1000	0.0236	[103]
SiO_2_ NFAs^*e*^	/	0.0256	[140]
SiO_2_ NFAs^*f*^	1100	0.0389	[141]
ZrO_2_-SiO_2_ NFMs^*g*^	1100	0.034	[159]
MMT@ZrO_2_-SiO_2_ NFMs	1000	0.026	[164]
SNF/SA membranes^*h*^	/	0.021	[166]
SiO_2_-SSNF^*i*^	/	0.025	[167]
SiO_2_ NFAs^*j*^	1100	0.02327	[168]
SiO_2_ NFAs^*k*^	1000	0.02196	[169]
ZrO_2_-SiO_2_ NFAs^*l*^	1100	0.0268	[170]
ZrO_2_-SiO_2_ NFAs^*m*^	1100	0.024	[171]

^*a*^SiO_2_/C nanofibrous aerogels (NFAs); ^*b*^SiO_2_ NFAs were prepared via freeze-drying method by combining SNFs with AlBSi matrices; ^*c*^SiO_2_ NFAs were prepared by integrating SNFs and Si–O-Si bonding networks; ^*d*^SiO_2_ composite nanofibers (CNFs); ^*e*^SiO_2_ NFAs were fabricted using SNFs as the matrix and SiO_2_ sol as high-temperature nanoglue; ^*f*^SiO_2_ NFAs were developed via lamellar stacking method through combining SNFs with AlBSi matrices; ^*g*^ZrO_2_-SiO_2_ nanofibrous membranes (NFMs); ^*h*^SiO_2_ nanofiber/SiO_2_ aerogel (SNF/SA) membranes; ^*i*^SiO_2_/SnO_2_ nanofibers reinforced flexible SiO_2_ aerogel composites (SiO_2_-SSNF); ^*j*^SiO_2_ NFAs were synthesized by using SNFs and SiO_2_ nanoparticle aerogels as the matrix and SiO_2_ sol as the high-temperature nanoglue; ^*k*^SiO_2_ NFAs were prepared by in situ assembly of nanoporous SiO_2_ aerogels on a cellular structured SiO_2_ fibrous framework; ^*l*^ZrO_2_-SiO_2_ NFAs were fabricated via lamellar stacking method by combining flexible ZrO_2_-SiO_2_ nanofibers with SiO_2_ sol solution; ^*m*^ZrO_2_-SiO_2_ NFAs were designed by integrating SiO_2_ granular aerogels and ZrO_2_-SiO_2_ nanofibers

#### Sound Absorption

Electrospun SNFs not only play an important role in the field of heat insulation, but also show great potential in the application of sound absorption and noise reduction. Specifically, their highly porous structure and good thermal stability lay a solid foundation for maintenance of outstanding sound absorption performance in a relatively confined environment, especially in vehicles and rooms. Zong et al. developed a simple and effective method to prepare flexible SiO_2_ nanofibrous sponges for highly noise absorption [172]. The hierarchical structured sponge was composed of flexible electrospun SNFs and reduced graphene oxide (rGO), consisting of open cells, closed cell walls, and entangled networks (Fig. 9d). It was found that with the increase in rGO loading, the coverage region of rGO networks on the SiO_2_ nanofibrous cell walls progressively increased, leading to the decrease in nanofibrous cell walls connectivity in sponge. In other words, the nanofibrous cell walls structure of sponge underwent a series of changes from the first open cell walls to the semi-open cell walls and then to the closed cell walls (Fig. 9e). It is conceivable that the internal structure of a sponge has a decisive effect on its sound-absorbing performance. As is shown in Fig. 9f-g, further studies have confirmed that the closed cell walls formed by a certain amount of rGo loading was the best for sound absorption performance, and the sponge possessed highest airflow resistance (6.7 × 10^5^ Pa s m^−2^) and noise reduction coefficient (NRC value of 0.27). This was because the closed cell wall structures in sponge can completely block noise propagation and make the noise dissipation need to go through a longer path, thus achieving the purpose of effective energy dissipation (Fig. 9e).

Although the ceramic nanofibrous sponge described above had excellent sound absorption properties, its application in extreme environments was seriously threatened because the loaded rGO was difficult to exist stably in high-temperature oxidation environments. Therefore, Cao and co-workers designed and prepared elastic SiO_2_ nanofibrous aerogels, which were assembled by immobilizing hexagonal boron nitride (h-BN) flakes on electrospun SNFs through freeze-shaping technology [147]. In the freeze-shaping process, h-BN flakes were covered by sol networks and adhered to SNFs to form extraordinary multi-scale 3D structure. It should be noted that the introduction of h-BN not only increased the multiple reflection and friction of sound waves by creating a more tortuous hierarchical structure, thus consuming more energy, but also the good thermal conductivity of h-BN helped dissipate sound energy in the form of friction heat generation. Benefits from a well-designed structure, the as-prepared nanofibrous aerogel had competitive sound absorption performance with NRC value of 0.59. At the same time, as a high-temperature resistant inorganic material, h-BN is capable of remaining stable at relatively higher temperatures oxygenated environment compared to rGO, so this kind of nanofibrous aerogel has great application potential.

Although some progress has been made in the development of electrospun SNFs materials for fire proof and sound absorption in recent years, some problems still need to be considered. For example, it is true that the sound absorption performance of materials in low frequency band has been improved to some extent, but there is still a lot of room for improvement compared with the absorption coefficient in high frequency band. Therefore, it is still an urgent problem to prepare high-efficiency sound-absorbing materials in all frequency range. In addition, oxide ceramic nanofibrous materials are generally hydrophilic and easy to absorb moisture, which brings great challenges to maintain the sound absorption properties of materials with long-term stability [173]. The proper solution of these problems will further enhance the practical application level of electrospun SNFs sound-absorbing materials.

#### Toxics Degradation

Because of its stable chemical properties, high strength, and easy processing, electrospun SNFs are often combined with other inorganic nanofibrous materials to develop novel functional materials, especially when they are used to degrade some toxic substances to ensure people’s health, the results often show unexpected surprises. To take a typical example, Liao et al. elaborately designed a honeycomb-like 3D nanofibrous aerogels composed of electrospun SNFs and Zr(OH)_4_@PVB nanofibers, in which Zr(OH)_4_ nanoflakes grew vertically and uniformly on the interconnected nanofibrous skeleton (Fig. 9h) [174]. The electrospun SNFs in aerogels played an important role in the construction of mechanically strong and structurally stable nanofibrous cellular framework. It was also proved that insufficient SNFs led to the collapse of the resulting nanofibrous aerogel structure, which unable to serve for subsequent applications in the form of self-supporting 3D blocks. Moreover, the Zr(OH)_4_ nanoflakes were evenly and stably embedded into the PVB nanofibrous template, which significantly expanded the surface area and provided abundant active sites for catalyzing chemical warfare agents. The dimethyl methylphosphonate (DMMP) was selected as a simulated nerve agent to analyze the possible mechanism of its degradation by aerogel: firstly, the coordination between P = O and Zr^IV^ cations, followed by nucleophilic substitution of hydroxyl group on the surface of nanoflakes (Fig. 9i). Further study found that when the aerogels contained 80 wt% Zr(OH)_4_@PVB nanofibers, the aerogels had the highest content of Zr(OH)_4_ and also exhibited the best catalytic effect and 99% conversion within 20 min (Fig. 9j). The extraction chromatography also confirmed the catalytic degradation of DMMP by as-prepared aerogel, and the final degradation product was methanol (Fig. 9k).

In addition to chemical warfare agents in war, there are also volatile organic compounds in daily life, such as formaldehyde, toluene, and xylene, and prolonged exposure to these compounds can be extremely harmful to the human body. Cui et al. fabricated soft SiO_2_-TiO_2_ nanofibrous membranes by electrospinning, on which MnO_2_ nanoparticles were in situ deposited [175]. It was found that the loading capacity of MnO_2_ nanoparticles increased gradually with the increase in the number of synthesis cycles. Because the MnO_2_ nanoparticles were fixed stably on the surface of the nanofibers, it has excellent catalytic oxidation effect on formaldehyde gas. The formaldehyde removal efficiency of the prepared sample was nearly 100% within 20 min, and the sample still showed a formaldehyde removal efficiency of 91.57% after 5 cycles of use. Moreover, Zhan and colleagues designed SiO_2_-doped mesoporous TiO_2_ nanofibers and verified their excellent photocatalytic performance through degrading gaseous toluene under ultraviolet light [176]. The results show that SiO_2_-TiO_2_ composite nanofibers showed a toluene degradation efficiency of 90.6% under the appropriate SiO_2_ doping, which was far superior to pure TiO_2_ nanofibers (69.6%) and commercial Degussa P25 (70.5%). The strong coupling effect between the doped SiO_2_ and the TiO_2_ may reduce the photoexcitation level and reduce the difficulty of toluene degradation removal. At the same time, these catalysts were used for the removal of organic pollutants in the form of electrospun nanofibrous membranes, which effectively avoided the drawbacks of conventional powder catalysts such as easy agglomeration and difficult recovery.

Some progress has been made in the degradation of toxic substances by electrospun SNFs, especially when they were compounded with other functional nanomaterials, showed the robust synergistic effects that greatly improve the overall properties. However, in the face of increasingly complex living environment, all kinds of harmful substances to human health need to be considered, so it is particularly necessary to improve the simultaneous degradation performance of materials to a variety of toxic substances. In addition, for toxics in different states, such as gas, liquid, and aerosol, the structure of materials needs to be elaborately designed to meet the use requirements of different scenarios.

### Health Care

#### Tissue Engineering

The natural bone extracellular matrix (ECM) is composed of 60–70 wt% inorganic components and 10–30 wt% organic components and presents a network structure of nanofibers [177]. Electrospun SNFs not only can highly simulate the hierarchical structure of ECM, but also have biocompatibility and low toxicity, showing greater osteogenic potential in bone tissue engineering applications [178]. Allo and co-workers firstly synthesized electrospun biomaterials by the combination of biodegradable polycaprolactone (PCL) and bioactive glasses (BGs), in which BGs were ternary inorganic phase including SiO_2_, CaO, and P_2_O_5_ [179]. The tertiary BGs used in study mimicked calcified tissue in bone, while the biodegradable PCL mimicked the nanofibrous collagen, a structure highly mimicked bone ECM that may provide better conditions for bone tissue regeneration. Unfortunately, no quantitative characterization of the electrospun SNFs for bone tissue engineering applications was available in the publication. Toskas et al. employed chitosan (CTS), containing a small amount of PEO, and SiO_2_ precursor sol to prepare composite biomaterials for bone tissue regeneration by electrospinning technology [180]. It was found that the nanofibrous membranes were beneficial to the adhesion and diffusion of osteoblasts, and the formation of hydroxyapatite was accelerated by impregnating modified simulated body fluids (SBF) after adding calcium ions. These hybrid nanofibers, composed of biocompatible polymers and SiO_2_, took full advantage of these two materials to successfully create effective biomaterials for bone tissue engineering.

The abovementioned works did not involve calcination of the as-spun nanofibers, and it is known that the conventional preparation of ceramic nanofibers inevitably required calcination to remove organic components. Therefore, different calcination conditions had a great impact on whether the obtained nanofibers were suitable for bone tissue engineering. Sakai and colleagues found in their study that apatite particles with a diameter of 10 μm were formed on the SNFs without calcination after soaking in SBF solution for 1 week [181]. Furthermore, with the increase in the calcination temperature, the diameter of the particles gradually decreases, and even no apatite particles were formed after calcination at 800 °C for 3 h. The study also proved that the apatite particles formed on SNFs could effectively promote the osteogenic differentiation of pre-osteogenic cells, which also explained from the side that the obtained nanofibers by high-temperature calcination were not conducive to the application of bone tissue engineering. In addition to preparing biomaterials with a single inorganic component, Wang and co-workers prepared electrospun SiO_2_-TiO_2_ hybrid nanofibers with different SiO_2_ content and evaluated their osteogenic potential [182]. The results showed that the resulting SiO_2_-TiO_2_ nanofibrous membrane enhanced the osteogenic differentiation of mesenchymal stem cells (MSC), especially when the average diameter of the nanofibers was larger and the crystallinity of the nanofibrous membrane was higher.

The electrospun SNFs prepared above were generally 2D nanofibrous membranes with pore sizes of several microns, which were adverse to the transport of nutrients and metabolites and difficult to provide enough space for cell growth. Wang et al. developed a 3D ceramic nanofibrous scaffold assembled by electrospun SNFs with CTS as the bonding site via freeze-drying technology [183]. The resulting scaffolds exhibited good elasticity, rapid deformation recovery, and excellent fatigue resistance and directly induced osteogenic differentiation of human MSC in vitro. The study also confirmed that the superelastic scaffolds could adapt to the mandibular defects in rabbits and promoted the bone formation of the calvarial defect in rats. In addition, the 3D scaffolds with SNFs gradients possessed significant functional effects, resulting in stiffness gradients spatially and differentiation of human MSC into chondrocytes and osteoblasts.

**Fig. 10:**
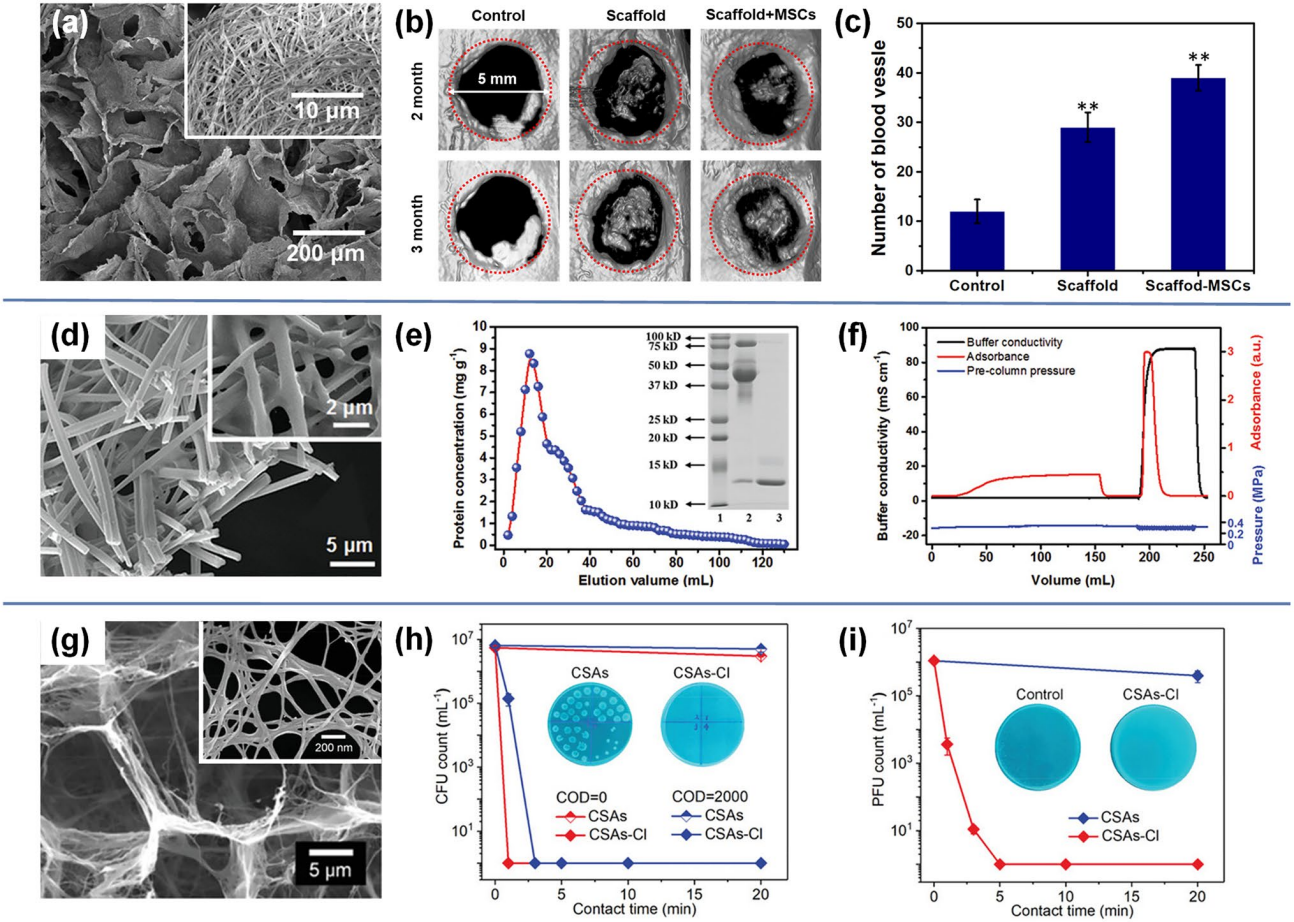
**a** SEM image of the SiO_2_-CaO nanofibrous scaffold [184]. **b** 3D reconstructed micro-CT images of rat cranial bone defects [184]. **c** Quantitative analysis of regenerated blood vessels [184]. Copyright 2019, American Chemical Society. **d** SEM image showing the nanofibrous cell wall of the aerogel [187]. **e** Dynamic elution curve of the aerogel-packed column after extracted lysozyme from egg white. The insets are the corresponding gel electrophoresis analysis of the obtained elution [187]. **f** The obtained chromatogram of the prepacked aerogel column [187]. Copyright 2019, Wiley–VCH. **g** SEM image of the cage-like structured aerogel [193]. **h** Bactericidal kinetics of the as-prepared aerogels against E. Coli [193]. **i** Biocidal assay against bacteriophage of the as-prepared aerogels [193]. Copyright 2021, Wiley–VCH

Although the prepared SNFs/CTS scaffold had good biocompatibility and bone repair performance, only SiO_2_ showed limited biomineralization activity and was difficult to accelerate bone regeneration in osteoporosis. Wang and co-workers further prepared bioactive glass (SiO_2_-CaO) nanofibers and combined them with CTS as cross-linking agent to synthesize 3D nanofibrous scaffolds [184]. It was found that the scaffolds composed of SiO_2_-CaO nanofibers and CTS had superior mechanical properties. In addition, the scaffolds possessed continuous nanofiber-assembled cell walls, which were structurally highly consistent with the natural ECM (Fig. 10a). The excellent bone repair capability of obtained scaffolds was verified by cranial defect model in osteoporotic rats. Micro-CT images of the bone defect site 2 and 3 months after surgery showed that the host bones in the control group were slightly mineralized from the periphery inward, while the defect edge and center of the as-prepared scaffolds were significantly mineralized (Fig. 10b). When theses scaffolds were used to repair osteoporotic calvarial defects in a rat, which showed significant improvement in new bone formation and vascular remodeling of osteoporotic bone defects. As is demonstrated in Fig. 10c, a lot of capillaries were observed in both at the edges and in the center of defects implanted with scaffolds and MSC-loaded scaffolds. This flexible nanofibrous scaffold, assembled by using flexible bioactive glass nanofibers as the construction blocks, could undergo elastic deformation and adapt to irregular shaped bone defects and then achieve perfect adaptation to the defects by self-deploying behavior. This strategy will provide a pathway for the development of the next generation of nanofibrous bone scaffolds, especially for irregularly shaped bone defects associated with osteoporosis.

As we can see, electrospun SNFs employed in bone tissue engineering have made progress in recent years. Bone tissue is a kind of dynamic load-bearing connective tissue, which plays a crucial role in maintaining normal life activities of organisms. Therefore, in the development of bone tissue engineering scaffold materials, it should not only be considered as the carrier of seed cells and growth factors, but also ensure that the scaffold has enough mechanical strength to support the growth of tissue. In addition, sufficient attention should be paid to the degradation of scaffolds. Reasonable matching of scaffold degradation rate and new bone formation rate is of great significance for obtaining the best bone repair effect.

#### Protein Separation

The electrospun SNFs have good physical and chemical stability, easy modification, and large surface area-to-volume ratio, which are the main reasons for their application in protein adsorption separation. Matthew and co-workers synthesized the SiO_2_/PVP composites nanofibrous membranes and used them for binding/elution of plasmid deoxyribonucleic acid and bovine serum protein [185]. The experimental results showed that the nanofibrous membranes had an effective binding ability to protein molecules, which was closely related to the surface charge and pH value of protein molecules. Although this publication does not provide a quantitative characterization of the adsorption and separation efficiency of the nanofibrous membranes for protein, it demonstrated that electrospun SNFs were an important tool for efficient isolation and delivery of proteins. Moreover, Zhu et al. prepared a novel ordered mesoporous SiO_2_/C composite nanofibers via electrospinning and followed by carbonization [186]. Benefiting from the highly ordered mesoporous structure, high specific surface area and pore volume of the resulting composite nanofibers, the materials showed excellent adsorption performance for the extraction and prefractionation of peptides from human serum.

Although the introduction of these electrospun SNFs based on 2D nanofibrous membrane promoted the actual performance of protein separation, their dense structure led to problems such as high resistance of protein molecule adsorption and mass transfer and slow liquid penetration velocity, which limited the full play of structural advantages of nanofibers. Fu and colleagues developed a highly carboxylated nanofibrous aerogels consisted of flexible electrospun SNFs and a functional polymer cladding layer [187]. The as-prepared aerogel presented a honeycomb-like regular and interconnected nanofibrous framework, and PVA layers were observed to be stably coated on the cell walls of the framework (Fig. 10d). Due to the negatively charged carboxyl ligands on the carboxylated PVA layer, the cell walls could selectively adsorb positively charged proteins in the solution, thus endowing the carboxylated nanofibrous aerogel with good protein adsorption and separation ability. Further study proved that the aerogel could directly adsorb and extract lysozyme from egg white solution, and the concentration of lysozyme in eluting solution was up to 9 mg mL^−1^ (Fig. 10e). When the carboxylated nanofibers aerogel were applied in the protein separation chromatography and purification system, the chromatographic curves showed that the aerogel had the advantages of high flux and low flow resistance (Fig. 10f). Moreover, Fu and co-workers fabricated a highly phosphorylated nanofibrous aerogel through a combination of freeze drying and a non-damaging surface modification technique [188]. In situ phosphorylation modification realized stable cross-linking of the aerogel and formed stable adhesion structure between flexible electrospun SNFs as the construction blocks and effectively improved its mechanical properties. The aerogel exhibited excellent static (3.3 × 10^3^ mg g^−1^) and dynamic (1.8 × 10^3^ mg g^−1^) protein adsorption capacity, with processing flux, was up to 1.5 × 10^4^ L h^−1^ m^−2^ only driven by the buffer’s own gravity (~ 1 kPa).

It can be found that most of these materials were organic/inorganic hybrid nanofibrous materials, in which the organic components will inevitably swell in the long-term practical application. This will seriously affect the morphology, pore structure, and mechanical stability of the material, which will significantly reduce the adsorption and separation performance of the material for protein. In addition, the electrospun SNFs applied in the field of protein separation in current studies basically relied on surface modification or in situ blending methods and pay little attention to the importance of nanofibrous intrinsic structure. Therefore, continuous efforts should be made in the relationship between composition controlled nanofibrous microstructure and their performance.

#### Antibiosis

Because of their excellent biocompatibility, good structural tunability, and easy surface modification, electrospun SNFs have impressive antibacterial performance. Wan et al. prepared composite nanofibrous membranes by combining electrospinning and surface modification technology [189]. The Ag nanoparticles with an average diameter of 50 nm were evenly and densely distributed on the SNFs modified by polydopamine. The results showed that the resulting nanofibrous membranes showed good antibacterial activity against Gram-negative bacteria *E. coli* and Gram-positive bacteria *S. aureus* due to the presence of Ag nanoparticles. Furthermore, Liu and co-workers developed a superhydrophilic N-halamine/SiO_2_ nanofibrous membranes through the combination of electrospinning technique and followed chlorination [190]. The obtained membranes exhibited the integrated properties of high active chlorine content, excellent re-chlorination performance, and strong bactericidal ability, and only 10 mg nanofibrous membranes could kill 3 × 10^8^ CFU mL^−1^ *E. coli* and *S. aureus* within 3 min. In addition to grafting modification on the surface of nanofibrous membrane, Shan et al. designed and prepared a new type of C/SiO_2_ nanofibrous antibacterial material supported by Co nanoparticles [191]. The metal Co nanoparticles with monodisperse distribution on the surface and inside of porous C/SiO_2_ nanofibrous membranes could effectively activate peroxymonosulfate and rapidly produce a large amount of reactive oxygen species, which could inactivate *E. coli* and *S. aureus* with a 7 log reduction within 3 min. The membranes also showed excellent dynamic bactericidal performance and could achieve high bactericidal efficiency of 99.99999% with flux up to 3.4 × 10^4^ L m^−2^ h^−1^ only under gravity drive. It is worth mentioning that a novel spray sterilizer based on nanofibrous membranes was designed, which could sterilize the solid surfaces conveniently and efficiently.

Although these membranes showed good antibacterial effects, their antibacterial function gradually decreased with the depletion of antibacterial ingredients. At the same time, the narrow and unconnected pore structure of the nanofibrous membrane makes it easy for bacteria to accumulate in the pore, resulting in a sharp decrease in the treatment flux. Wang et al. prepared a superelastic nanofibrous aerogel with rechargeable bactericidal properties by electrospun SNFs and functional Si–O-Si bonding networks [192]. The excellent structural stability and persistent bactericidal activity of the obtained aerogels were attributed to the Si–O-Si networks composed of rechargeable N-halamine groups. Therefore, the aerogels had excellent bactericidal activity (6 log reduction against *E. coli* and *S. aureus*) and could effectively sterilize the bacteria-containing sewage with ultra-high throughput (5.76 × 10^4^ L m^−2^ h^−1^). In addition, Wang and colleagues also developed a superflexible nanofibrous aerogel with antibacterial and antiviral functions targeting harmful microorganisms such as bacteria and viruses that may exist in the public health environment [193]. The aerogel was composed of electrospun SNFs, bacterial cellulose (BC) nanofibers, and hydrophobic Si–O-Si binder. The SNFs formed the primary nanofibrous framework, and the BC nanofibers (with an average diameter of one order of magnitude lower than that of SNFs) formed the secondary nanonet on the SiO_2_ framework (Fig. 10g). The Si–O-Si binder gave aerogels strong structural stability and hydrophobicity, and N-halamine biocides grafted aerogels with renewable antibacterial and antiviral activity. As is demonstrated in Fig. 10h-i, thanks to the numerous integrated advantages of the cage-like nanofibrous aerogel, it not only showed high filtration efficiency (> 99.97%) and low pressure drop (189 Pa) toward PM_0.3_, but also showed excellent antibacterial (6 log reduction against *E. coli* within 3 min) and antiviral activity (6 log reduction against bacteriophage within 5 min). These positive results indicate that the prepared aerogel can be used as a scalable antibacterial and antiviral air filter, which means that it has great potential for health care.

At present, most of the researches on electrospun SNFs in antibacterial field focus on inactivating bacteria existing in liquid environment, and the killing of pathogens and microorganisms in air environment is relatively rare. The continuing spread of the COVID-19 in the world since 2020 has alarmed us and highlighted the importance of developing integrated nanofibrous filters with broad-spectrum biocidal activity as well as high efficiency fine particulate filtration performance. In addition, special attention should be paid to the recycling properties of these antibacterial materials in order to reduce the pressure on resources and the environment. Therefore, the future research of such materials should consider more convenient and easily scalable preparation methods combined with energy-saving high-performance regeneration means.

### Water Treatment

#### Pollutant Removal

**Fig. 11:**
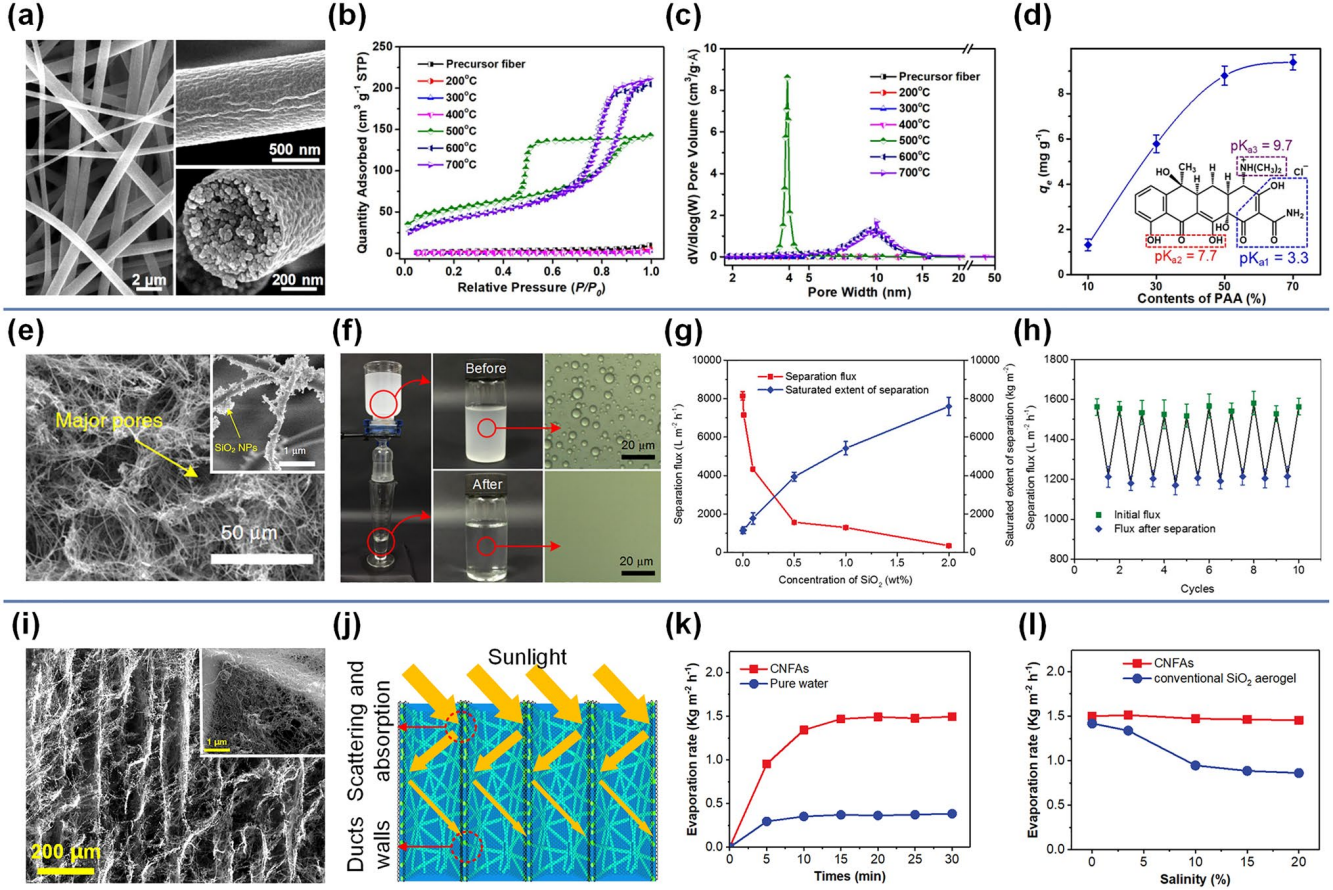
**a** SEM images of mesoporous SNFs [194]. **b** Nitrogen adsorption–desorption isotherms of mesoporous SNFs at various calcination temperature [194]. **c** The corresponding pore size distribution curves [194]. **d** Adsorption performance of relevant mesoporous SNFs [194]. Copyright 2021, Elsevier. **e** SEM image showing open cell geometry of the aerogels. The inset is the corresponding magnified SEM image revealing SiO_2_ nanoparticles anchored on the nanofibers [203]. **f** Separation device of water-in-oil emulsions and the microscopic photographs of emulsions before and after separation [203]. **g** Separation flux and saturated extent of separation of nanofibrous aerogels [203]. **h** Changes of the flux and flux recovery over 10 cycles [203]. Copyright 2015, American Chemical Society. **i** SEM image showing cellular architecture of the aerogel. The inset is the magnified SEM image displaying the CNTs networks in nanofibrous framework [208]. **j** Schematic illustration of multiple scattering and absorption of light [208]. **k** Evaporation rate versus solar light irradiation time under 1-sun irradiation [208]. **l** The evaporation rate of as-prepared aerogels in different salinity of brine under 1-sun irradiation [208]. Copyright 2020, Wiley–VCH

Due to their good chemical stability, high specific surface area and strong structural tunability, electrospun SNFs are ideal materials for removing many pollutants in wastewater, including antibiotics, heavy metal ions, organic dyes, and phosphates. Shan and colleagues prepared mesoporous electrospun SNFs with excellent flexibility, high specific surface area, and large pore volume [194]. By adjusting the content of polymer in the spinning solution, the phase separation degree of liquid jets was affected during the electrospinning process, and thus the nanofibers morphologies and pore structures were controlled. As presented in Fig. 11a, after calcination at 600 °C, the surface of the nanofibers was rough and wrinkled obviously. Some isolated particles and visible pores can be observed from the section diagram. Further analysis of the pore structure of the obtained nanofibers by calcination at different temperatures showed that the adsorption capacity of the samples was almost negligible below 400 °C, indicating that there were almost no pores in the nanofibers. However, when the temperature was increased to 600 °C, the specific surface area increased to 147.76 m^2^ g^−1^, the pore volume increased to 0.317 cm^3^ g^−1^, and the mesopore size concentrated at 10 nm (Fig. 11b-c). Therefore, the nanofibrous membranes showed good adsorption performance for tetracycline hydrochloride, and the maximum adsorption capacity was 9.4 mg g^−1^ (Fig. 11d). These results were consistent with the total pore volume of the corresponding nanofibers, indicating that increasing the pore volume was beneficial to the adsorption of antibiotics. Especially, further analysis indicated that the synergistic effect of hydrogen bond and electrostatic attraction between the electrospun SNFs and tetracycline hydrochloride was responsible for such excellent adsorption performance. In addition, this highly porous electrospun SNFs had also been extended to many heavy metal ion adsorption applications, including Pb^2+^, Cu^2+^, Cd^2+^, and Cr^4+^, exhibiting good ion adsorption performance [195–198].

Through the functional modification of the electrospun SNFs, it had high catalytic degradation effect on organic pollutants in water. Wang et al. designed and fabricated a hierarchical electrospun SNFs on which CuO-ZnO nanosheets were deposited [117]. The prepared nanofibrous membranes possessed large pore volume and mesoporous size, and the maximum adsorption capacity for Congo red reached 141.8 mg g^−1^. At the same time, the introduction of CuO-ZnO nanosheets also enabled the fiber membrane showed good catalytic activity to 4‑nitrophenol and could degrade more than 96% of the pollutants within 90 s. Moreover, Shi and colleagues developed CuFe_2_O_4_ nanostructure functionalized electrospun SNFs [199]. Benefiting from uniformly distributed CuFe_2_O_4_ particles, high specific surface area, and large pore volume, the membrane presented significantly improved Fenton-like catalytic degradation activity. The resultant nanofibrous membranes possessed good catalytic performance for methylene blue, which could be degraded by 96% in 20 min, and the removal rate could reach 0.148 min^−1^. In addition, there were also some reports about the removal of phosphate and mercaptan from water, demonstrating the advantages of the application of electrospun SNFs for the adsorption of various pollutants [200, 201].

According to these reported works, electrospun SNFs have shown great potential in removing pollutants from water. While these advances are welcome, it is also important to recognize that there is still a lot of research to be done on this issue. For example, the water environment is increasingly severe and complex, facing the threat of metal ions, pesticides, dyes, radioactive elements, and other types of pollutants, so it is particularly necessary to improve the efficient removal ability of materials for a variety of pollutants in the complex water environment. In addition, most of the materials currently prepared are applied in the form of 2D nanofibrous membranes, which will inevitably encounter problems of limited adsorption capacity and treatment flux. Therefore, the development of new-type 3D nanofibrous materials for the removal of pollutants in water is another topic that is particularly worthy of research.

#### Oil–Water Separation

Electrospun SNFs have unique advantages in the treatment of oily wastewater due to their high chemical stability, interconnected pore channel, high porosity, and regulable surface properties. Li et al. synthesized the hierarchical nanofibrous membranes composed of SiO_2_ nanofibers/nanobeads via the combination of electrospinning and electrospraying [126]. Thanks to their good wettability and hierarchical pore structure, the nanofibrous membranes possessed excellent performances in the separation of oil-in-water emulsions. Especially for surfactant stabilized oil-in-water emulsions, the membranes showed a separation efficiency of 98.8% and a permeability flux of 2237 L m^−2^ h^−1^. Furthermore, Zhang and co-workers developed a taro leaf-inspired nanofibrous membranes by anchoring BiOBr microspheres on SiO_2_/PANI nanofibrous substrates [202]. Due to the high porosity and submicron pore size of the resulting nanofibrous membrane, it can efficiently separate various oil-in-water emulsions, showing high separation flux (a maximum value of 6140 L m^−2^ h^−1^) and high separation efficiency (total organic carbon content less than 5 mg L^−1^). Notably, the BiOBr/PANI heterojunction structure and 3D PANI conductive networks endowed the nanofibrous membrane visible light induced self-cleaning properties. In addition, Zhang et al. also prepared another intriguing nanofibrous membrane by constructing periodic knots on electrospun SNFs and further encapsulating the shell of PANI [118]. The membrane could achieve efficient separation of oil-in-water emulsions, especially due to the synergistic effect of positive charge and spindle-knot structure, and the filter cake could be removed during the separation process, thus guaranteeing the stability of water flux.

In any case, the abovementioned nanofibrous membranes used for oil–water separation will face the bottleneck problems of low separation flux and limited separation efficiency. Si et al. reported a superelastic and superhydrophobic nanofibrous aerogel through combining electrospun nanofibers and freeze-drying technology [203]. The electrospun SNFs components in the aerogel fully ensured the stability of its overall shape and structure, and SiO_2_ nanoparticles were deposited on the nanofiber surface to further enhance the nanoscale roughness of the hierarchical aerogel (Fig. 11e). As a test of the separation performance of aerogel to oil-in-water emulsion, oil quickly penetrated through aerogel when surfactant stabilized oil-in-water emulsion just touched the surface of aerogel, while water remained in the upper layer of aerogel. The results showed that the water content in the filtrate was less than 50 ppm, which signified that the purity of the oil collected after separation by aerogel was up to 99.995% (Fig. 11f). The study also found that with the increase of SiO_2_ nanoparticles concentration, although the separation flux of aerogel decreased, its separation saturation capacity increased significantly. This may be related to the fact that over time, the continuous accumulation of water droplets would change the channel from hydrophobic to hydrophilic and eventually blocked the oil transportation routes. In particular, the aerogel loaded with 2 wt% SiO_2_ nanoparticles showed an admirable flux of 350 ± 45 L m^−2^ h^−1^ but an astonishing saturation capacity of 7612 ± 480 kg m^−2^ (Fig. 11g). As shown in Fig. 11h, after repeated separation experiment for 10 cycles, the aerogel can still efficiently separate oil–water emulsion, highlighting that aerogel also had excellent anti-pollution performance. In addition, other organic/SiO_2_ hybrid nanofibrous aerogels have been developed using similar methods and their applications in the field of oil–water separation have been explored [204, 205].

In fact, oil and water mixtures in real industrial production and daily life are characterized by complex composition, usually containing proteins, organic dyes, heavy metal ions, and so on. Therefore, the design of multifunctional separation membrane has important practical significance for the comprehensive treatment of oil–water mixture. It is a challenging project to equip the separation membrane with the functions of oily wastewater treatment, heavy metal ion adsorption, and organic matter degradation, and perhaps the coupling design of multilayer nanofibrous membrane is a feasible solution. In addition, when high viscosity crude oil emulsion is separated by high-pressure drive, the pore structure is not stable enough because of the insufficient bond among the nanofibers, which seriously affects the separation efficiency of the emulsion. Therefore, how to improve the bond strength between nanofibers as much as possible without compromising the inherent high porosity and pore connectivity of nanofibrous materials is a work worthy of further study.

#### Solar Desalination

Solar evaporator is an effective method to treat highly saline water with clean and renewable energy and realize seawater desalination. Electrospun SNFs have good chemical durability and high porosity, which facilitates evaporation of water vapor from the pores between the nanofibers. Huang and colleagues fabricated core-sheath structure and amphiphobicity SNFs via coaxial electrospinning technology [206]. The SiO_2_ nanoparticles on the nanofiber surface significantly increased the local roughness and improved the robustness of wetting resistance. It is found that in the presence of surfactant, the resulting membrane had a good potential for seawater desalination. Furthermore, Sun et al. designed and prepared a novel nanofibrous membrane composed of forest-like carbon nanotubes (CNTs) deposited on deposited on porous electrospun SNFs [207]. The engineered CNTs were assembled into a dense, rough, and porous interfacial structure with excellent moisture resistance to water in the air. Because of these characteristics, the as-prepared nanofibrous membranes showed stable water vapor flux and excellent salt repulsion in thermal driven desalination experiments.

In the actual desalination process, the surface salinity of the evaporator often increases with evaporation. We also know that electrospun nanofibers are usually assembled into relatively dense 2D membranes, whose pore connectivity and quantity are not ideal, impeding salt transport from the surface of the evaporator to the outside. Dong and co-workers fabricated elastic SiO_2_ nanofibrous aerogel which was then deposited on the nanofibrous framework by simple CNTs impregnation coating [208]. The obtained nanofibrous aerogel exhibited vertically arranged cells and porous cell walls, and CNTs were tightly encapsulated on the cell walls (Fig. 11i). The performance of solar evaporator is highly dependent on the absorption effect of the evaporator. By virtue of this ingenious design, light entered the cells, and when light hits the aerogel surface, most of the light was absorbed by CNTs deposited on the cell walls. Other scattered light was absorbed almost completely after hitting the cell wall several times (Fig. 11j). It was found that the evaporation rate of the as-prepared aerogel could reach 1.5 kg m^−2^ h^−1^ within 15 min, which was in the same level with most materials at present. However, it should be emphasized that the evaporation rate of the aerogel prepared has little change in the treatment of water with high salinity, while the evaporation rate of the conventional SiO_2_ aerogel as the control group decreased significantly, proving that the well-designed aerogel could effectively transport salt and prevent salt crystallization (Fig. 11k-l). In addition, inspired by the microstructure of reed leaves, Dong et al. prepared biomimetic hierarchical SiO_2_ nanofibrous aerogel with parallel-arrayed cells and hydrophobic surfaces [209]. Thanks to the advantages of this bionic structure, the resulting aerogel could work stably in high concentration of brine (saturated concentration, 26.3%) under 6 sun, demonstrating its robust salt tolerance. Specifically, due to its high light absorption efficiency of 94.8%, the evaporation rate of the as-prepared aerogel was 1.25 kg m^−2^ h^−1^ under 1 sun irradiation.

Solar desalination is a sustainable and low-energy strategy to alleviate the water crisis, and several studies have demonstrated the contribution of electrospun SNFs in this field. Although many researchers have made great efforts to improve the efficiency of solar desalination, it still needs to continue to advance the research work considering the practical application. How to effectively avoid salt crystallization in the process of solar desalination is still an unavoidable topic, which will have a vital impact on the long-term, efficient, and stable operation of evaporator. In addition, the recovery and utilization of latent heat of water vapor condensation is expected to relieve the high dependence on solar illumination conditions, thus further improving the efficiency of water production.

## Conclusions and Perspectives

In this review, we have summarized the advances in electrospun SNFs, covering the rational structure design from solid to core-sheath, hollow, porous, hierarchical, aligned, and 3D-assembled structure, and relevant synthetic strategies including coaxial electrospinning method, sacrificial template method, *in situ* growth method, freeze-drying method and so on. Moreover, the mechanical behavior of electrospun SNFs was also discussed, with emphasis on the origin of superior flexibility and the effective means of mechanical reinforcement. By virtue of their intriguing characteristics such as regulable morphology, high porosity, modifiable surface, and chemical stability, electrospun SNFs demonstrated tremendous potential in many areas, especially in the fields of physical protection (e.g., thermal insulation, sound absorption, and toxics degradation), health care (e.g., tissue engineering, protein separation, and antibiosis), and water treatment (e.g., pollutant removal, oil–water separation, and solar desalination).

Although the great progress has been made, there are still many challenges in the design, preparation, and application of electrospun SNFs, and more work needs to be continued in the future. Some viewpoints are put forward here, which are expected to play a constructive role in promoting the rapid development of this field.

1. At present, most reports focus on core-sheath, porous, hierarchical structure of electrospun SNFs, and more attractive nanostructures are yet to be developed. For example, the widely reported spider-web-like 2D nanonets in electrospun polymer materials. These nanonets possess a highly porous structure with ultrafine diameters (10 ~ 40 nm) and small pore sizes, which hold great potential in energy, filtration, and protection applications [210]. Different from the preparation of polymer nanofibers, the preparation of SNFs involves a mixture of SiO_2_ precursor sol and polymer solution spinning and then calcination. Therefore, the distinctions of spinning dope properties and the influences of calcination process should be concerned in the preparation of SNFs with 2D nanostructured networks.
2. As a promising new material, electrospun SiO_2_ nanofibrous aerogel has been widely studied in recent years. As we have seen, several methods (e.g., freeze-drying method and lamellar stacking method) have been developed to prepare electrospun SiO_2_ nanofibrous aerogel, and its application in many areas has been explored. However, it should be acknowledged that the current method of preparing SiO_2_ nanofibrous aerogel is generally cumbersome, time-consuming, and energy intensive, especially involving the acquisition of staple slurry from electrospun SNFs and subsequent freeze drying. Recently, a novel approach to fabricate nanofibrous aerogel using 3D reaction electrospinning has opened a new avenue for us [211]. The key to the success of this approach is to regulate the gelation rate of inorganic sol jet so as to realize the precise control of jet shape.
3. The current tensile strength of flexible SNFs membranes is normally less than 5 MPa, while the compressive stress of SiO_2_ nanofibrous aerogels prepared by freeze-drying method is generally less than 20 kPa. These materials are still difficult to meet the requirements of mechanical properties for some specific applications. It is an urgent problem to further improve the mechanical properties of SNFs. The key to solve this problem lies in the fabrication of strong and tough single SiO_2_ nanofiber. On this basis, building strong adhesion structure between nanofibers or improving the orientation of nanofibers might be effective strategies to enhance the mechanical properties of SNFs. In addition, it is of great significance to establish theoretical models of the mechanical relationship between single nanofiber and nanofibrous assemblies in the future work, which could guide researchers to design SNFs with higher strength and better toughness.
4. In the future, advanced applications of electrospun SNFs will go far beyond the scope of our discussion, and the existing wealth of results will continue to inspire us to create new materials. There is no need to elaborate on the characteristic advantages of SiO_2_ material itself, and more creativity can be generated from the aspects of structure, interface and multi-scale assembly. For example, the construction of ordered hierarchical pores in SNFs will bring a significant driver for hydrogen storage, molecular separation, and gas capture. In addition, combining the fertile surfaces of SNFs with other specific materials promises infinite possibilities in the fields of sensing, electromagnetic shielding, and flexible energy. In short, more substantial and effective applications of electrospun SiO_2_ nanofibrous materials are worthy of further study, and the road to the application library will never be limited.
5. The large-scale manufacture of electrospun SNFs is the cornerstone to realize their commercial value. Currently, most of the synthesis methods of functional SNFs (e.g., in situ growth method, liquid-phase reaction method, and freeze-drying method) are limited to laboratory research and are not suitable for large-scale production. Although some polymer nanofibers could be manufactured on an industrial scale, the impact of different spinning solution systems on mass production is significant, especially for spinneret devices. Some auxiliary means can be well utilized, such as high-speed airflow and rotary centrifugation, which are expected to significantly improve manufacturing efficiency. In addition, the continuity, uniformity, and efficiency of conventional multi-needle electrospinning are seriously affected because of the electric field disturbances between multiple needles and the faster gelation rate of SiO_2_ precursor sol. Therefore, needleless electrospinning is equipped with the advantages of simple construction, uniform electric field, and high efficiency, which is expected to become the main force of mass production of electrospun SNFs in the future.

Challenge goes together with chance, and difficulty coexists with hope. Even though there are many troubles to be faced in the future, there is no doubt that electrospinning technology is still the most powerful weapon to prepare advanced SNFs. In the future, the rapid development of SNFs will be realized through the cross-fusion of multidisciplinary knowledge of materials science, material mechanics, and mechanical engineering. We firmly believe that the design, manufacture, and application of electrospun SNFs will have an exciting and bright future with continuously push.
